# Yes-associated protein plays oncogenic roles in human sporadic colorectal adenomas

**DOI:** 10.1093/carcin/bgaf007

**Published:** 2025-02-20

**Authors:** Lei Fan, Xingyi Guo, Mary K Washington, Jiajun Shi, Reid M Ness, Qi Liu, Wanqing Wen, Shuya Huang, Xiao Liu, Qiuyin Cai, Wei Zheng, Robert J Coffey, Martha J Shrubsole, Timothy Su

**Affiliations:** Division of Epidemiology, Department of Medicine, Vanderbilt Epidemiology Center, Vanderbilt-Ingram Cancer Center, Vanderbilt University Medical Center, 2525 West End Avenue, Nashville, TN 37203, United States; Division of Epidemiology, Department of Medicine, Vanderbilt Epidemiology Center, Vanderbilt-Ingram Cancer Center, Vanderbilt University Medical Center, 2525 West End Avenue, Nashville, TN 37203, United States; GRECC, Department of Veterans Affairs, Tennessee Valley Healthcare System, 1310 24th Avenue S., Nashville, TN 37212, United States; Department of Pathology, Vanderbilt University Medical Center, 1211 Medical Center Drive, Nashville, TN 37232, United States; Division of Epidemiology, Department of Medicine, Vanderbilt Epidemiology Center, Vanderbilt-Ingram Cancer Center, Vanderbilt University Medical Center, 2525 West End Avenue, Nashville, TN 37203, United States; GRECC, Department of Veterans Affairs, Tennessee Valley Healthcare System, 1310 24th Avenue S., Nashville, TN 37212, United States; Division of Gastroenterology, Department of Medicine, Vanderbilt University Medical Center, 1211 Medical Center Drive, Nashville, TN 37232, United States; Center for Quantitative Sciences and Department of Biostatistics, Vanderbilt University School of Medicine, 1211 Medical Center Drive, Nashville, TN 37232, United States; Division of Epidemiology, Department of Medicine, Vanderbilt Epidemiology Center, Vanderbilt-Ingram Cancer Center, Vanderbilt University Medical Center, 2525 West End Avenue, Nashville, TN 37203, United States; GRECC, Department of Veterans Affairs, Tennessee Valley Healthcare System, 1310 24th Avenue S., Nashville, TN 37212, United States; Department of Breast Surgery, The Second Hospital of Shandong University, 247 Beiyuan Street, Jinan, Shandong 250031, China; Center for Quantitative Sciences and Department of Biostatistics, Vanderbilt University School of Medicine, 1211 Medical Center Drive, Nashville, TN 37232, United States; Division of Epidemiology, Department of Medicine, Vanderbilt Epidemiology Center, Vanderbilt-Ingram Cancer Center, Vanderbilt University Medical Center, 2525 West End Avenue, Nashville, TN 37203, United States; GRECC, Department of Veterans Affairs, Tennessee Valley Healthcare System, 1310 24th Avenue S., Nashville, TN 37212, United States; Division of Epidemiology, Department of Medicine, Vanderbilt Epidemiology Center, Vanderbilt-Ingram Cancer Center, Vanderbilt University Medical Center, 2525 West End Avenue, Nashville, TN 37203, United States; GRECC, Department of Veterans Affairs, Tennessee Valley Healthcare System, 1310 24th Avenue S., Nashville, TN 37212, United States; Division of Gastroenterology, Department of Medicine, Vanderbilt University Medical Center, 1211 Medical Center Drive, Nashville, TN 37232, United States; Cell and Development Biology, Vanderbilt University, 1211 Medical Center Drive, Nashville, TN 37232, United States; Division of Epidemiology, Department of Medicine, Vanderbilt Epidemiology Center, Vanderbilt-Ingram Cancer Center, Vanderbilt University Medical Center, 2525 West End Avenue, Nashville, TN 37203, United States; GRECC, Department of Veterans Affairs, Tennessee Valley Healthcare System, 1310 24th Avenue S., Nashville, TN 37212, United States; Division of Epidemiology, Department of Medicine, Vanderbilt Epidemiology Center, Vanderbilt-Ingram Cancer Center, Vanderbilt University Medical Center, 2525 West End Avenue, Nashville, TN 37203, United States; GRECC, Department of Veterans Affairs, Tennessee Valley Healthcare System, 1310 24th Avenue S., Nashville, TN 37212, United States

**Keywords:** colorectal adenoma, Hippo-YAP, Wnt pathway, oncogene, biomarker

## Abstract

The role of Hippo-Yes-associated protein (YAP) in human colorectal cancer (CRC) presents contradictory results. We examined the function of YAP in the early stages of CRC by quantitatively measuring the expression of phospho-YAP^S127^ (p-YAP) and five APC-related proteins in 145 sporadic adenomas from the Tennessee Colorectal Polyp Study, conducting *APC* sequencing for 114 adenomas, and analyzing YAP-correlated cancer pathways using gene expression data from 326 adenomas obtained from Gene Expression Omnibus. The p-YAP expression was significantly correlated with YAP expression (*r* = 0.53, *P* < .0001) and nuclear β-catenin (*r* = 0.26, *P* = .0018) in adenoma tissues. Both p-YAP and nuclear β-catenin were associated with *APC* mutations (*P* = .05). A strong association was observed between p-YAP overexpression and advanced adenoma odds (OR = 12.62, 95% CI = 4.57–34.86, *P* trend < .001), which persisted after adjusting for covariates and biomarkers (OR = 12.31, 95% CI = 3.78–40.10, *P* trend < .0001). P-YAP exhibited a sensitivity of 77.4% and specificity of 78.2% in defining advanced versus nonadvanced adenomas. Additionally, synergistic interaction was noted between p-YAP positivity and nuclear β-catenin on advanced adenomas (OR = 16.82, 95% CI = 4.41–64.08, *P* < .0001). YAP-correlated genes were significantly enriched in autophagy, unfolded protein response, and sirtuin pathways showing predominantly pro-tumorigenic alterations. Collectively, YAP plays an oncogenic role in interacting with Wnt as well as other cancer pathways within human sporadic adenomas. P-YAP could be a potential biomarker for human high-risk sporadic adenomas.

## Teaser

YAP activation in human colorectal adenoma was significantly correlated with *APC* mutations, Wnt activation, and advanced adenoma odds. Furthermore, YAP may interact with Wnt/β-catenin, autophagy, unfolded protein response, and sirtuin pathways in the early stage of human colorectal carcinogenesis.

## Graphical Abstract

**Graphical Abstract:**
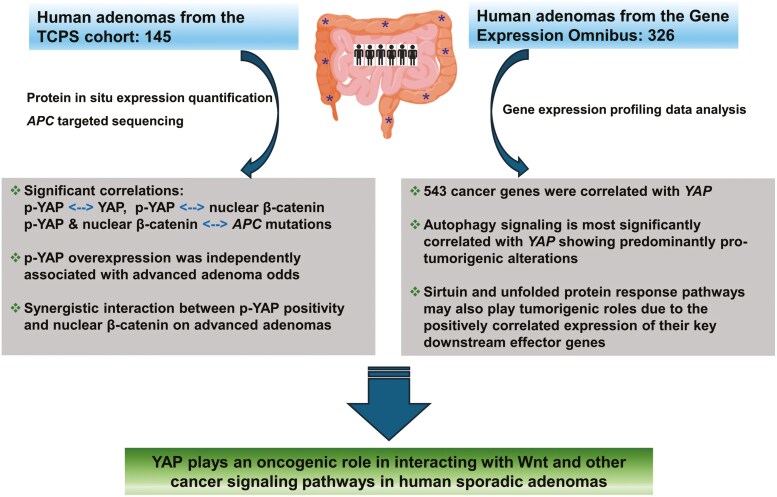

## Introduction

Colorectal cancer (CRC) is the second most deadly cancer in the USA [1]. Approximately 80% of CRC cases occur sporadically, without a positive family history or penetrant germline mutations [2]. The best way to prevent sporadic CRC is through early detection and removal of adenomatous polyps via colonoscopy. These precancerous tumors carry somatic mutations and can develop into cancer over time, from one to more than twenty years, depending on the accumulation of genetic and epigenetic alterations of more driver genes and the local colonic environment [3–5]. Current guidelines for postpolypectomy surveillance suggest varying time intervals based on initial colonoscopy findings such as location, number of polyps, and tumor size [6,7]; this can lead to both over- and under-surveillance. Therefore, it is crucial to investigate biomarkers that could better identify individuals at high risk of tumor recurrence or progression for more precise prevention strategies. Despite the high heterogeneity of CRC at both the clinical and molecular levels [8], microsatellite instability in tumor cells and mutations in KRAS and BRAF have been shown to have prognostic and predictive significance for CRC patients [2,8]. However, there are hitherto no comparable biomarkers for the earlier stages of colorectal carcinogenesis [9–11].

Yes-associated protein (YAP) is a downstream transcriptional coactivator in the Hippo signaling pathway. Activation of YAP and its coregulator TAZ (YAP/TAZ) have either oncogenic roles [12,13] or antitumor effects [14–16]. Previous studies revealed conflicting results of YAP in human CRC, showing either tumor-promoting [17–19] or tumor-suppressive effects [20,21]. This discrepancy may be due to YAP’s function as a co-effector molecule that interacts with other signaling regulators to modulate pathways positively or negatively depending on tissue type, cell context, Hippo-independent regulatory molecules, and various stages of cancer [22]. A study on familial adenomatous polyposis (FAP), an inherited condition, found that nearly all human adenomas exhibited YAP activation following APC deficiency, indicating that YAP is crucial for the development of these adenomas in FAP patients [23]. Yet, research into YAP’s role in sporadic human adenomas remains limited.

This study aims to investigate the potential oncogenic role of YAP and its interaction with Wnt and other cancer signaling pathways in the early stages of human colorectal tumorigenesis and to explore potential early biomarkers of high-risk sporadic adenoma. We recruited a cohort of patients with sporadic colorectal adenoma and performed targeted sequencing on the APC gene along with in situ quantitative detection of phosphor-YAP^S127^ (p-YAP), N-terminal APC protein (APCn), and other key regulatory molecules of Wnt pathway (β-catenin) [23], G-protein signaling (Asef1/2) [24,25], and APC-correlated transcriptional coregulators (i.e. C-terminal binding protein (CtBP) and microtubule-associated protein RP/EB family member 1 (EB1)) [26–28]. Moreover, we analyzed gene expression data from human adenoma tissues found in the Gene Expression Omnibus repository to identify genes and functional pathways correlated with YAP.

## Materials and methods

### Study samples

The adenoma tissue samples used in this study were from the Tennessee Colorectal Polyp Study (TCPS), a colonoscopy-based case–control study conducted in Nashville, Tennessee [29]. Eligible participants aged 40–75 were identified from patients scheduled for colonoscopy at Vanderbilt University Medical Center and Nashville Veterans Affairs Medical Center. Excluded were those with a known family history of hereditary CRC syndromes, a history of inflammatory bowel disease, any previous colectomy, and any diagnosis of adenomas on previous colonoscopy or surgical resection, or any history of cancer other than nonmelanoma skin cancer. Eligible participants had at least one polyp found during the enrollment colonoscopy (baseline). After the enrollment colonoscopy, cases were followed for future colonoscopy to determine the occurrence of metachronous adenoma from retrieval of procedure and pathology reports. The Vanderbilt University Institutional Review Board, the Veterans’ Affairs Institutional Review Board, and the Veterans’ Affairs Research and Development Committee approved the study. In this study, a total of 145 baseline adenoma tissue samples, including small adenomas (62 cases, ≤4 mm), medium-size adenomas (30 cases, 4 to <10 mm), and large adenomas (50 cases, ≥10 mm) were selected randomly from the TCPS tissue repository. Among the 145 participants, metachronous (diagnosed more than 6 months after the baseline tumor) and synchronous (diagnosed within 6 months of the tumor) adenomas were present in 67 and 78 cases, respectively. To evaluate normal expression patterns of selected biomarkers, formalin-fixed paraffin-embedded (FFPE) normal rectal tissues from 22 de-identified nontumor patients provided by the Tissue Morphology Subcore of the Digestive Disease Research Center at Vanderbilt were used. The diagnosis of all tissue samples was confirmed by a centralized review of a research pathologist (T.S.) and a gastrointestinal pathologist (M.K.W.). The *in vitro* tumor size of the small early adenomas was measured under microscopy using the BioQuant Life Science imaging software (v.13.5.60 ME).

### Immunohistochemistry

**Figure 1:**
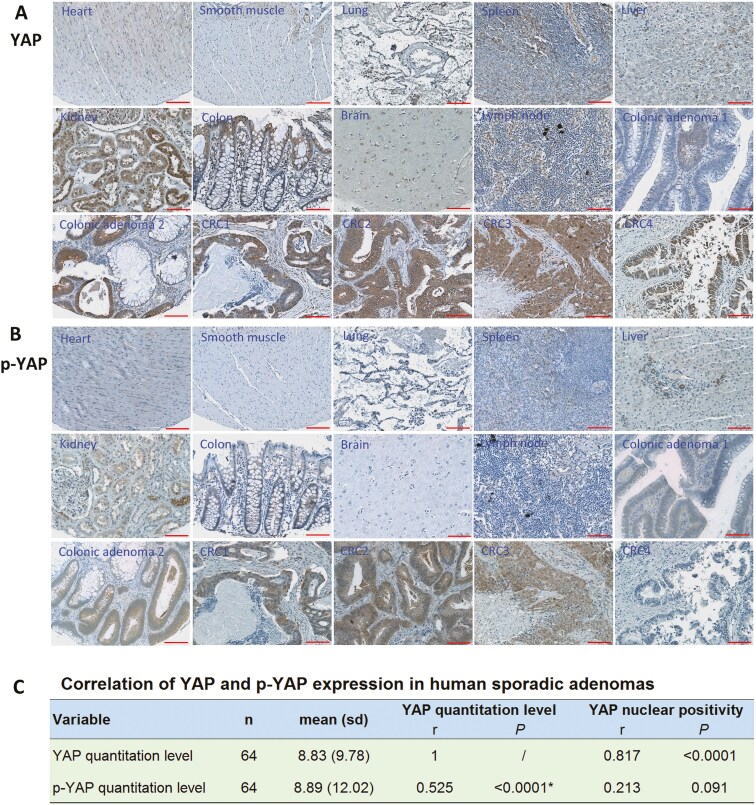
Selection and validation of YAP antibodies using TMA control and human adenoma tissues. (a) The YAP expression levels were elevated in CRC, some adenomas, and normal kidney, colon, and placenta tissues. (b) The expression levels of p-YAP were lower than that of YAP and showed negative staining in most normal tissues, including the colon. Scale bars: 100 µm at 10× magnification. (c) In a pilot of 64 adenomas (>4 mm in size), the YAP expression was closely correlated with p-YAP expression (*r* = 0.525, Pearson correlation coefficients) and YAP nuclear positivity (*r* = 0.817, Spearman correlation coefficients). Nuclear positively stained cells were scored semiquantitatively as: 0, no staining; 1, 1%–10%; 2, 11%–30%; 3, >31%.

Standard immunohistochemical staining for CtBP, p-YAP, EB1, and β-catenin was conducted following the DAKO Envision™ kit protocol (DAKO, Cat# K4011) with a Dako LV-1 Autostainer System (Dako Colorado Inc., USA). We first constructed a tissue microarray (TMA) block for primary antibody validation, staining protocol optimization, and quality control. The control TMA included 10 normal human tissues (kidney, liver, lung, brain, spleen, breast, colon, prostate, lymph node, and heart), 11 cancer (CRC × 6, breast cancer × 2, lung cancer × 2, metastatic CRC × 1) and 4 adenoma tissues. Using the control TMA slides, we compared the staining patterns of anti-YAP (1:250, Cat# HPA070359, Sigma Aldrich) and antiphosphor-YAP^S127^ (1:800, p-YAP, Cat# 13008, Cell Signaling) in various normal and tumor tissues, showing that YAP expression exhibited in cancers (CRC, breast cancer and lung cancer), colonic adenoma and several types of normal tissues (colon with mucosa and smooth muscle, kidney, liver, placenta, spleen, lymph node, lung, and brain) (Fig. 1a); in contrast, p-YAP expression was weaker than YAP in cancer and adenoma tissues and undetectable in the most normal tissues except a weak expression in the convoluted tubes of kidney and the small bile ducts of liver (Fig. 1b). To explore the correlation between YAP and p-YAP in the human adenoma tissues, we performed a pilot study using 64 adenoma samples (> 4 mm in size), showing a significant correlation between YAP and p-YAP expression (*r* = 0.53, *P* < .0001. Fig. 1c). Based on the preliminary data, we selected the anti-p-YAP antibody as a potential biomarker to process for all adenoma samples. The other primary antibodies used in this study were rabbit anti-CtBP (C-Terminus) (1:200, Cat# LS-C355983, LifeSpan BioSciences), rabbit anti-EB1 (1:200, Cat# HPA003600-100UL, Sigma-Aldrich), and rabbit anti-CTNNB1 (β-catenin) (1:400, Cat# HPA029160, Sigma-Aldrich). The antibody specificity was validated by comparing the staining patterns in the various TMA control tissues with the data from the Human Protein Atlas (https://www.proteinatlas.org/about#the_human_protein_atlas) (Supplementary Fig. 1a).

The APC N-terminal binding protein Asef and APCn protein (amino acids 1–226 of APC) were detected using a highly sensitive sequential fluorescence double-staining method incorporated with tyramide signal amplification (TSA) technique [30] (Supplementary Fig. 1b). In brief, after antigen retrieval using a pressure cooker in pH6.0 citrate buffer and blocking steps with hydrogen peroxide and normal goat serum, the slides are incubated with mouse anti-APCn antibody (1:500, EMD Millipore, Cat# MAB3785) for 1 hour at room temperature (RT), the secondary antibody (Super Picture HRP Polymer Conjugate Broad Spectrum, Life Technologies, Cat# 878963) for 30 min at RT, and TSA-FITC (1:100, PerkinElmer Opal 3-plex Kit, NEL791001KT) for 3 min at RT. After rinsing the slide, we strip the previous antibodies using the microwave heating method [31], repeat blocking steps, and add rabbit anti-Asef antibody (1:400, NBP1-88857, Novus Biologicals) for 1 hour at RT, followed by the above secondary antibody for 30 min and TSA-Cy3 for 5 min at RT. Coverslip the slides with ProLong Gold Antifade Reagent with DAPI (Cat# P36935, Invitrogen) and store the slides at 4ºC in sealed boxes. The TMA control slides were stained in parallel with each batch of study samples for quality control. We observed consistent staining results by comparing TMA reference tissues.

### Imaging quantification and semiquantification

The quantification of four biomarkers (CtBP, p-YAP, EB1, and β-catenin) detected by standard immunohistochemistry (IHC) was performed using computer-assisted automatic imaging software to obtain reliable and reproducible results following three steps (Supplementary Fig. 2). First, images were edited using the threshold tools of BioQuant Life Science imaging software (BioQuant, Nashville, TN) to exclude the stromal area and any unwanted tissue folding area or crush artifacts. Second, the brightfield image was converted into a darkfield pseudo-coloring image using free segmentation software ilastik 1.3.2 [32,33]. Last, the automatic imaging quantification of the converted pseudo-coloring images was performed by developing measurement pipelines for each biomarker using CellProfiler software v3.1.5 [34,35]. Because of interoperability between ilastik and CellProfiler software packages [36], CellProfiler can run an ilastik machine-learning algorithm from within a CellProfiler pipeline for high throughput image processing and analysis without tedious manual manipulation. The quantifications of APCn and Asef detected by fluorescence double staining were performed similarly, without converting images using ilastik software. The semiquantification of nuclear β-catenin accumulation in tumor cells was performed according to our previous study’s four-category (0–3) method [37].

### *APC* targeted sequencing

Targeted sequencing data for *APC* was successfully obtained from 114 baseline adenomas and 32 metachronous adenomas in this study. The FFPE tissue sections were macrodissected to limit to adenomatous tissue (one to five 10 μm slides depending on tumor size). The DNA isolation and purification were conducted using the QIAamp DNA FFPE Tissue Kit (QIAGEN, Cat#56404) protocol. A total of 50 μl (2 ng/μl) genomic DNA from each sample was used for exome sequencing of *APC* and other selected genes (not reported in this study). All primer development and next-generation sequencing were conducted by the collaborative Covance Genomics Laboratory (Redmond, WA), the drug development arm of LabCorp. The Illumina NextGen sequencing platform (GAIIx Sequencer) was used, and the sequencing depth was 500X.

### Bioinformatic exploration of *YAP*-correlated pathways in human adenomas

We comprehensively searched and downloaded gene expression data in human adenoma tissues (*n* = 326, after excluding 22 sessile serrated adenomas) from the Gene Expression Omnibus (https://www.ncbi.nlm.nih.gov/geo/; including GSE10714, GSE4183, GSE20916, GSE37364, GSE117606, GSE117607, GSE8671, GSE44076, GSE79462, GSE19963, GSE45270, GSE33113, GSE39582, GSE35896, GSE13067, GSE13294, GSE14333, GSE17536, GSE2109, GSE23878, GSE37892, GSE71187 and GSE76987). Multiple quality control steps were performed to standardize the data, including removing low-expressed genes, log2 transformation, and Robust Multiarray Average quantile normalization. The Spearman rank correlation between *YAP* and other coding genes was calculated using the normalized gene expression data. We identified 2,697*YAP*-correlated genes based on a threshold at *R*^2^ > 0.5 and further examined their functional enrichment in the gene function category and biological pathways using the Ingenuity Pathway Analysis (IPA) tool. The top five significant function categories and six biological pathways were analyzed and presented in this study.

### Statistical analysis

Differences between groups were analyzed using chi-square or Fisher’s exact test for categorical data and independent *t*-test (2 groups) or ANOVA (3 groups) for continuous data. When comparing biomarker expression of small or larger adenomas to the adjacent normal colonic mucosa, the paired Student’s *t*-test was performed. The correlations of the selected biomarkers were analyzed using Spearman correlation coefficients. All analyses were two-sided with a significance level of 5% and performed using SAS statistical software (v9.3).

## Results

### Characteristics of study participants

The demographic and clinicopathological parameters of the participants are shown in Table 1. There were no significant differences in age, sex, and race among the three adenoma groups except for tumor sizes. The tubulovillous histotype was more common in the large adenoma group (56.0%) than in the small (3.1%) and medium-sized (30.0%) groups (*P* < .001). As expected, high-grade dysplasia was only present in the large adenoma group. The 22 nontumor patients who were included as normal controls were younger (*P* < .001) and more frequently female (*P* = .001) than the three adenoma groups but without significant differences in race (*P* = .52).

**Table 1.** Demographic and clinicopathological parameters of adenoma patients from the Tennessee Colorectal Polyp Study.

Variables	Cases	Small (<4 mm)	Medium (4 to < 10 mm)	Large (>=10 mm)	*P* ^*1*^	*P* ^*2*^
	*n*	*n* = 65	*n* = 30	*n* = 50		
Age					.814	<.001
Mean (SD)	145	59.6 (6.2)	59.1 (7.0)	59.9 (6.5)		
Sex, *n* (%)					.238	.001
Male	94	42 (64.6)	16 (53.3)	36 (72.0)		
Female	51	23 (35.4)	14 (46.7)	14 (28.0)		
Race, *n* (%)^a^					.711	.52
Caucasian (white)	120	52 (80.0)	26 (86.7)	42 (87.5)		
African American (black)	18	11 (16.9)	3 (10.0)	4 (8.3)		
Other	5	2 (3.1)	1 (3.3)	2 (4.2)		
Tumor size					< .0001	
Mean (SD)	145	2.1 (0.6)	6.6 (1.8)	14.3 (6.9)		
Histotype, *n* (%)					< .0001	
Tubular	106	63 (96.9)	21 (70.0)	22 (44.0)		
Tubulovillous/villous	39	2 (3.1)	9 (30.0)	28 (56.0)		
Dysplasia, *n* (%)					.0003	
Low	137	65 (100.0)	30 (100.0)	42 (84.0)		
High	8	0 (0)	0 (0)	8 (16.0)		
Adenoma recurrence, *n* (%)					.3953	
No	78	39 (60.0)	15 (50.0)	24 (48.0)		
Yes	67	26 (40.0)	15 (50.0)	26 (52.0)		

*P* ^1^: Small versus medium versus larger adenoma comparison used the chi-square test for categorical data and the Kruskal–Wallis test for continuous data. *P*^*2*^: Normal rectum versus adenoma.

^a^Two cases of adenoma patients missed race data.

### *In situ* expression of biomarkers in adenoma and nontumor colorectal tissues

**Figure 2:**
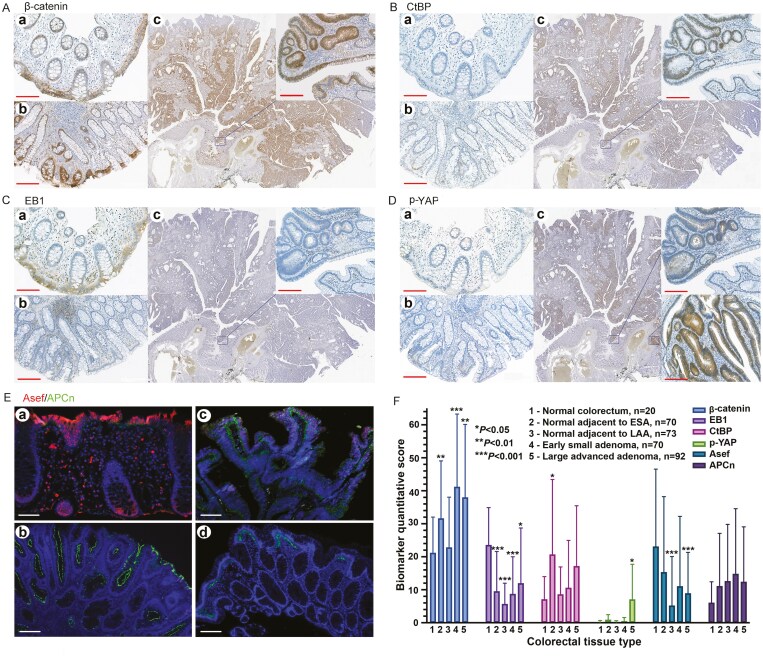
Expression of p-YAP and other biomarkers in adenoma, adjacent nontumor, and normal colorectal tissues. (A) p-YAP expression is weak in the normal rectal mucosa (a) and slightly increased in the small adenoma and adjacent nontumor mucosa (b). The larger adenoma (c) shows an elevated expression of p-YAP in the cytoplasm of tumor cells (the upper-right corner) but may appear in the nuclei in the strong positive area (the lower right corner). (B) The membranous β-catenin can be seen in the normal rectal epithelium (a) and the elevated expression is seen in small adenoma (b) and larger adenoma (c). The nuclear accumulation in the boundary area of the tumor and nontumor in the larger adenoma is shown in the upper-right corner. (C) CtBP is negative in the normal rectal epithelium (a), weakly expressed in small adenoma and nontumor mucosa (b), and strongly expressed in larger adenoma (c) with nuclear staining pattern. (D) Cytoplasmic EB1 signal is strong in the normal rectal epithelium (a) and decreased in small (b) and larger (c) adenomas and adjacent nontumor mucosa. (E) The immunofluorescent dual staining shows that Asef is strongly expressed in the normal rectal mucosa (a) but decreased in small (b) and larger (c) adenomas and adjacent nontumor mucosa (d). Inversely, APCn is weakly expressed in the normal rectal epithelium but increased in small and larger adenoma and adjacent nontumor mucosa. Scale bars: 100 µm at 10X magnification. (F) The quantitative data of biomarkers in adenomas and adjacent nontumor mucosa, using normal rectal tissue as reference.

The expression of cytoplasmic p-YAP was low or negative in the normal rectal tissue, small adenomas, and surrounding nontumor tissues, and significantly higher in medium/large (>4 mm) adenomas (*P* = .02) with cytoplasmic/nuclear staining pattern (Fig. 2A). The expression of other selected biomarkers is shown in Fig. 2B–F. The nuclear accumulation of β-catenin was found in 30/67 (44.8%) small adenomas, 13/32 (40.6%) medium-size adenomas, and 27/50 (54.0%) large adenomas, without statistically significant differences in frequencies (*P* > .05). The nuclear accumulation of β-catenin in normal rectal tissue was not found in this study.

### Intercorrelations of p-YAP with selected biomarkers

The correlation of the biomarker expression levels in human adenomas is shown in Table 2. The expression of p-YAP was significantly correlated with EB1 (*r* = 0.32, *P* < .0001), CtBP (*r* = 0.5, *P* < .0001), β-catenin expression levels (*r* = 0.38, *P* < .0001) and its nuclear accumulation (*r* = 0.26, *P* = .0018). The expression level and nuclear accumulation of β-catenin were correlated (*r* = 0.21 *P* = .01), and both were significantly correlated with CtBP (*r* = 0.52, *P* < .0001; *r* = 0.23, *P* = .01, respectively).

**Table 2.** Intercorrelation of selected biomarkers in human adenoma tissues^a^, *n* = 144.

Biomarkers	Nuclear β-catenin		β-catenin		EB1		CtBP		APCn		Asef	
	*r*	*P*	*r*	*P*	*r*	*P*	*r*	*P*	*r*	*P*	*r*	*P*
β-catenin	0.21	.01										
EB1	0.09	.28	0.1	.2214								
CtBP	0.23	.01	0.52	< .0001	0.15	.0775						
APCn	−0.07	.41	−0.17	.0398	0.13	.1069	−0.07	.3842				
Asef	−0.01	.89	−0.10	.2103	0.005	.9502	−0.008	.9266	0.57	< .0001		
p-YAP	0.26	.0018	0.38	< .0001	0.32	< .0001	0.5	< .0001	0.04	.6263	0.11	.182

^a^Spearman correlation coefficients.

### Association p-YAP and other biomarkers with clinicopathological parameters of adenoma

The p-YAP expression was strongly associated with the odds ratio (OR) of large adenomas (OR = 14.25, 95% CI = 3.32–61.18, *P* trend < .0001), villous growth pattern (OR = 58.71, 95% CI = 10.49–328.61, *P* trend < .0001) and advanced adenoma (OR = 12.62, 95% CI = 4.57–34.86, *P* trend < .0001) (Table 3). In models including all biomarkers with a *P*-value < .20 in unadjusted models, overexpression of p-YAP and CtBP were independently associated with OR of advanced adenomas (OR = 12.31, 95% CI = 3.78–40.10, *P* trend < .0001; OR = 5.27, 95% CI = 1.25–22.235, *P* trend = 0.023, respectively) (Supplementary Table 1). Nuclear accumulation of β-catenin in baseline adenomas was associated with OR of large adenoma, and villous growth pattern (if scored 3 versus 0, OR = 4.76, 95% CI = 1.06–21.31, *P* trend = .055; OR = 4.62, 95% CI = 1.11–19.20, *P* trend = .013, respectively).

**Table 3.** Association of biomarker expression with clinicopathological parameters of adenomas.

Genetic variables	Tumor size					Histotype			Advanced adenoma^a^			Metachronous adenoma		
	<4 mm	4 to <10 mm		≥10 mm		TA	TV/V	OR (95% CI)	No	Yes	OR (95% CI)	No	Yes	OR (95% CI)
	*n*	*n*	OR (95% CI)	*n*	OR (95% CI)	*n*	*n*		*n*	*n*		*n*	*n*	
β-catenin														
0.41–20.71	11	10	1.00 (ref)	12	1.00 (ref)	24	9	1.00 (ref)	17	16	1.00 (ref)	12	21	1.00 (ref)
20.71–43.44	22	8	0.48 (0.12, 1.89)	14	0.70 (0.20, 2.43)	33	11	1.23 (0.38, 4.04)	28	16	0.76 (0.26, 2.27)	29	15	0.28 (0.09, 0.85)
43.44–84.72	31	12	0.48 (0.13, 1.78)	23	0.75 (0.23, 2.49)	48	18	0.79 (0.25, 2.50)	38	28	0.75 (0.26, 2.13)	36	30	0.58 (0.20, 1.62)
*P* trend			0.2921		0.7028			0.6326			0.6073			0.4747
Nuclear β-catenin (3 versus 0)														
0	35	17	1.00 (ref)	23	1.00 (ref)	60	15	1.00 (ref)	46	29	1.00 (ref)	39	36	1.00 (ref)
1	13	4	0.65 (0.13, 3.27)	8	0.72 (0.20, 2.56)	17	8	4.27 (1.13, 16.10)	14	11	1.07 (0.34, 3.38)	14	11	0.58 (0.19, 1.76)
2	12	7	2.34 (0.61, 8.94)	7	1.50 (0.42, 5.43)	19	7	3.92 (1.07, 14.35)	17	9	1.34 (0.44, 4.10)	16	10	0.52 (0.17, 1.53)
3	5	2	1.64 (0.22, 12.33)	11	4.76 (1.06, 21.31)	10	8	4.62 (1.11, 19.20)	7	11	3.30 (0.87, 12.53)	9	9	0.71 (0.19, 2.68)
*P* trend			0.2782		0.0551			0.0129			0.1136			0.2955
Nuclear β-catenin (>0 versus 0)														
0	35	17	1.00 (ref)	23	1.00 (ref)	60	15	1.00 (ref)	46	29	1.00 (ref)	39	36	1.00 (ref)
1–3	30	13	1.42 (0.50, 4.07)	26	1.57 (0.63, 3.93)	46	23	4.24 (1.53, 11.72)	38	31	1.57 (0.68, 3.58)	39	30	0.58 (0.26, 1.31)
*P* trend			0.5096		0.338			0.0054			0.2894			0.1923
EB1														
0.02–2.91	21	12	1.00 (ref)	10	1.00 (ref)	36	7	1.00 (ref)	30	13	1.00 (ref)	24	19	1.00 (ref)
2.91–8.64	22	9	0.61 (0.18, 2.13)	17	1.32 (0.41, 4.23)	32	16	2.09 (0.64, 6.91)	26	22	1.31 (0.46, 3.71)	22	26	1.29 (0.48, 3.44)
8.64–82.9	21	9	0.79 (0.23, 2.72)	23	1.84 (0.59, 5.76)	37	16	2.02 (0.63, 6.49)	27	26	1.57 (0.57, 4.35)	31	22	0.76 (0.28, 2.02)
*P* trend			0.708		0.2998			0.2679			0.3871			0.5462
CtBP														
0–4.61	30	11	1.00 (ref)	14	1.00 (ref)	49	6	1.00 (ref)	40	15	1.00 (ref)	26	29	1.00 (ref)
4.61–15.32	17	11	1.58 (0.47, 5.38)	8	0.79 (0.22, 2.92)	27	9	2.20 (0.57, 8.41)	22	14	1.79 (0.59, 5.41)	18	18	0.88 (0.32, 2.43)
15.32–93.59	18	8	1.94 (0.54, 6.90)	27	4.64 (1.56, 13.80)	29	24	7.50 (2.35, 23.963)	22	31	4.99 (1.80, 13.81)	34	19	0.49 (0.20, 1.24)
*P* trend			0.2805		0.0066			0.0006			0.0021			0.1405
Asef														
0–0.88	27	2	1.00 (ref)	3	1.00 (ref)	29	3	1.00 (ref)	27	5	1.00 (ref)	13	19	1.00 (ref)
0.88–6.92	21	17	9.32 (1.67, 52.17)	28	21.04 (3.65, 121.27)	44	22	9.39 (1.72, 51.27)	31	35	11.94 (2.67, 53.40)	34	32	0.55 (0.19, 1.61)
6.92–98.18	17	11	8.93 (1.48, 53.77)	19	18.13 (2.98, 110.30)	33	14	7.33 (1.26, 42.51)	26	21	9.11 (1.92, 43.21)	31	16	0.29 (0.09, 0.92)
*P* trend			0.0154		0.0022			0.0565			0.0173			0.0327
p-YAP														
0–0.08	19	6	1.00 (ref)	7	1.00 (ref)	74	3	1.00 (ref)^b^	63	14	1.00 (ref)^b^	19	13	1.00 (ref)
0.08–0.69	35	5	0.41 (0.08, 2.05)	5	0.33 (0.08, 1.50)							25	20	0.77 (0.26, 2.27)
0.69–55.72	11	19	11.81 (2.50, 55.72)	37	14.25 (3.32, 61.18)	31	36	58.71 (10.49, 328.61)	21	46	12.62 (4.57, 34.86)	33	34	1.16 (0.42, 3.24)
*P* trend			0.0013		< 0.0001			< 0.0001			< 0.0001			0.6599
APCn														
0–2.31	19	8	1.00 (ref)	9	1.00 (ref)	25	11	1.00 (ref)	22	14	1.00 (ref)	17	19	1.00 (ref)
2.31–12.85	23	14	0.72 (0.19, 2.75)	25	2.20 (0.66, 7.38)	46	16	0.70 (0.23, 2.11)	33	29	1.47 (0.52, 4.16)	31	31	0.96 (0.35, 2.67)
12.85–98.55	23	8	0.31 (0.07, 1.31)	16	0.97 (0.27, 3.49)	35	12	0.63 (0.18, 2.18)	29	18	0.85 (0.28, 2.62)	30	17	0.45 (0.15, 1.36)
*P* trend			0.1056		0.8142			0.47			0.7201			0.1362

ORs (95% CI) were adjusted for age, sex, race, BMI, smoking status, exercise activities, family history of CRC or polyps, and education.

^a^Advanced adenoma is defined as adenomas that are ≥10 mm or show tubulovillous/villous histotype or high-grade dysplasia.

^b^The lowest and 2nd tertiles for p-YAP are combined due to small sample sizes.

**Figure 3:**
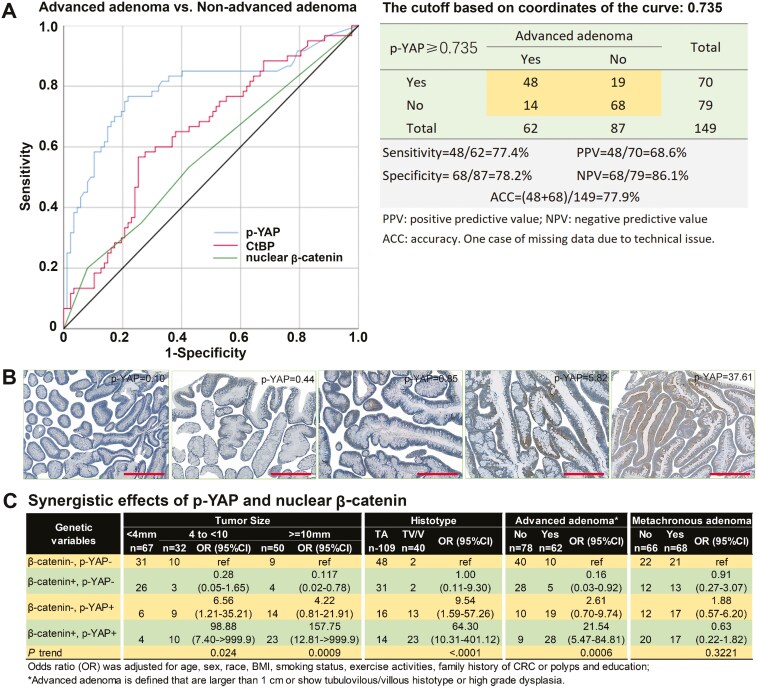
p-YAP as a biomarker differentiating advanced versus nonadvanced adenomas. (a) Comparison of the ROC curves showed that p-YAP (AUC=0.798, 95% CI: 0.719 - 0.877) was superior to CtBP (AUC=0.648, 95% CI: 0.558 - 0.739) and nuclear β-catenin (AUC=0.570, 95% CI: 0.475 - 0.666) in differentiating advanced adenomas. The best cutoff of p-YAP is 0.735 as a biomarker for advanced adenoma; (b) Example images of p-YAP negative (score < 0.735) and positive (score > 0.735) adenomas. Scale bars, 200 µm; (c) Synergistic relationship of nuclear β-catenin and p-YAP on tumorigenesis.

Figure 3a showed that p-YAP exhibited the highest sensitivity and specificity in defining advanced versus nonadvanced adenomas compared to CtBP and nuclear β-catenin by the AUC analysis [38]. The cutoff of 0.735 based on all coordinates of the p-YAP curve obtained the best sensitivity of 77.4% and specificity of 78.2%. According to conventional semiquantitative scoring criteria such as the Allred scoring system [37,39], a weak positive signal of p-YAP is equivalent to positive p-YAP (i.e. >0.735) in the IHC settings of this study (Fig. 3b). Finally, in exploratory analysis, we further analyzed the potential synergistic relationship of nuclear β-catenin and p-YAP positivity on tumorigenesis. ORs for advanced adenoma were highest for simultaneous positivity of nuclear β-catenin and p-YAP versus the other groups (OR = 26.36, 95% CI = 7.24–95.92, Fig. 3c).

### Association of *APC* mutations with p-YAP and selected biomarkers in human adenomas

**Figure 4:**
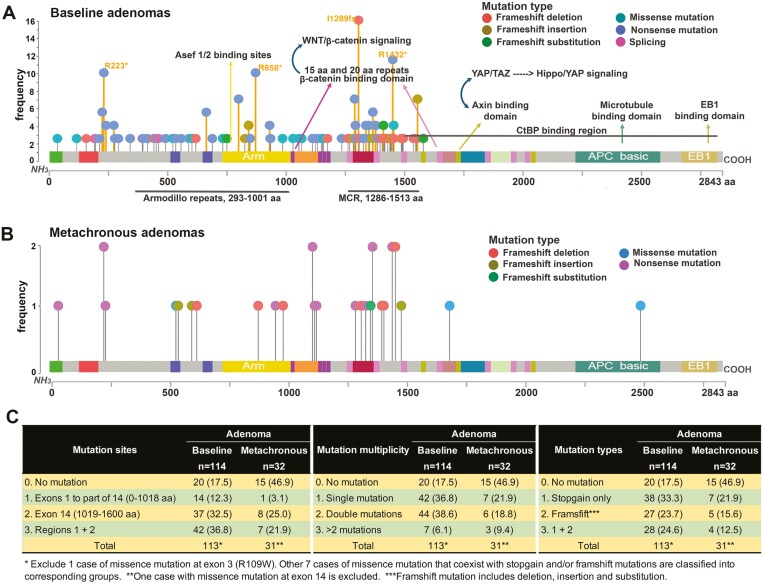
The frequency and distribution of APC mutations in baseline and metachronous adenoma tissues. (a) Distribution of APC mutations in the 114 baseline adenoma tissues, plotting by the MutPlot (https://bioinformaticstools.shinyapps.io/lollipop/) (b) Distribution of APC mutations in the 32 baseline adenoma tissues. (c) Baseline and metachronous adenomas showed similar APC mutation characteristics in mutation sites, multiplicity, and types (Fisher exact test, *P* > .05). *Frameshift mutation includes deletion, insertion, and substitution. **One case with missense mutation at exon 3 was excluded (TQ15338), and 7 other cases of missense mutation coexist with stopgain and/or frameshift mutations. ***One case with missense mutation at exon 14 was excluded.

The *APC* mutation was found in 94/114 (82.5%) cases of sporadic adenomas. All mutations fell within the regions that encode N-terminal 1–1600 amino acids with the highest mutation frequency in the mutation cluster region (MCR) followed by Armodillo repeats region (Fig. 4a). The pattern was similar in the metachronous adenomas (Fig. 4b). The most common mutation site was the region of exon 14 including the MCR (Fig. 4c). The most common mutation type was nonsense/stopgain mutation, followed by frameshift mutations. We did not find any statistically significant difference between synchronous and metachronous adenomas regarding APC mutation status (mutation frequency, site, multiplicity, and type) using all available APC mutation data of 200 samples (including 50 case-matched metachronous adenomas) from TCPS participants (*P* > .05, Supplementary Table 2). Further analysis revealed that APC mutation status was significantly associated with β-catenin nuclear accumulation and p-YAP expression (*P* = .054 and *P* = .05, respectively) (Supplementary Table 3) and significantly associated with larger tumor size (OR = 8.082, 95% CI = 1.93–33.89) but not with other adenoma features.

### *YAP*-correlated genes and signaling pathways in human colorectal adenoma

**Figure 5:**
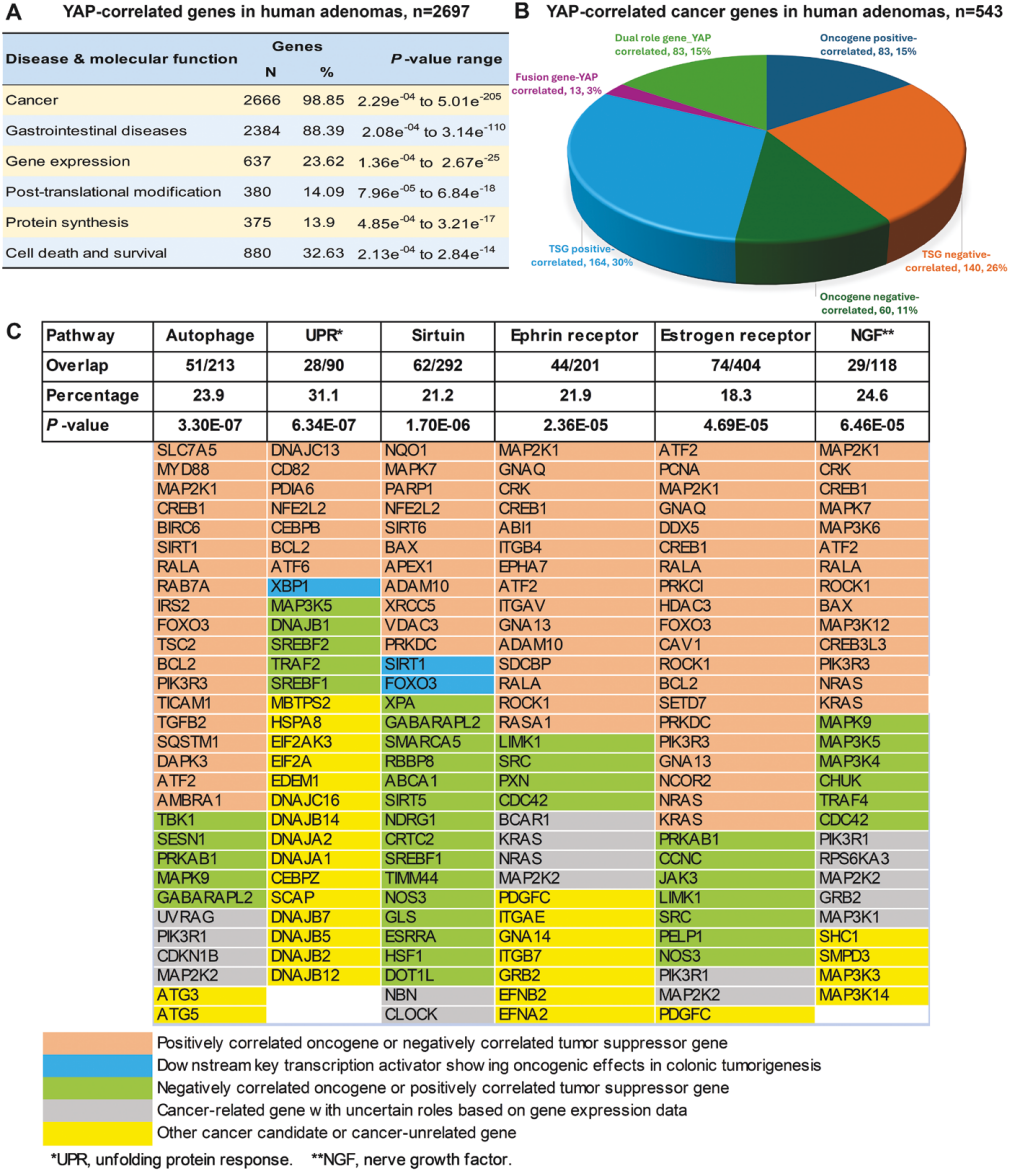
*YAP* potentially regulated genes and signaling pathways in human adenomas. (a) A total of 2697 genes were correlated with *YAP* gene expression and significantly enriched in molecular and cellular functions of gene expression, posttranslational modification, protein synthesis, and cell death and survival; (b) YAP-correlated cancer genes identified in this analysis. TSG, tumor suppressor gene; (c) The top six signaling pathways are enriched in the *YAP*-correlated genes.

The gene expression profiling analysis of 326 human adenomas from the Gene Expression Omnibus showed that 2697 genes either were positively (*n* = 1386) or negatively (*n* = 1311) correlated with *YAP* gene expression. These genes were significantly enriched in cancer and gastrointestinal diseases (*P* < .001 for both) revealed by IPA and were significantly enriched in molecular and cellular functions of gene expression, posttranslational modification, protein synthesis, and cell death and survival (*P* < 0.001 for all) (Fig. 5a). By comparing to our cancer gene database developed on Network of Cancer Genes (NGS6.0), UniProtKB, COSMIC, and NCBI annotated human cancer genes, we found 543 cancer genes out of 2697 genes, detailed in Fig. 5b. The top six signaling pathways enriched in the *YAP*-correlated genes were highlighted in Fig. 5c. Specifically, autophagy signaling is most significantly correlated with *YAP* (23.9% overlap, *P* = 3.30e^−7^). Sirtuin signaling and unfolded protein response pathways may also play tumorigenic roles due to the positively correlated expression of their main downstream effector genes, i.e. *SIRT1*, *FOXO3*, and *XBP1*, the latter was the top 4th of the most correlated upstream regulators related to *YAP* (*P* = 2.90e^−7^).

## Discussion

Our study found that p-YAP expression in the normal colorectal mucosa of nonadenoma patients was either negative or very weak, indicating low levels of YAP phosphorylation due to Hippo pathway regulation for normal tissue homeostasis. P-YAP expression slightly increased in small adenomas (less than 4 mm) and their unaffected colonic mucosa, but significantly elevated in larger adenomas (≥4 mm) and positively correlated with YAP expression. We discovered a significant correlation between p-YAP expression, *APC* mutations, and nuclear accumulation of β-catenin, suggesting an interrelationship between YAP deregulation and deficient APC/Wnt activation. Evidence showed that *APC* loss resulted in upregulation of both YAP and p-YAP through an enhanced IL-6 signal transducer which activates Src family kinases to augment YAP phosphorylation in human CRC cells [40]. Another research indicated that nuclear β-catenin/TCF4 complexes bind a DNA enhancer element within the first intron of the YAP gene driving its expression when Wnt/β-catenin signaling is aberrant in human CRC cells [41]. The abnormal increase of cytoplasmic YAP enhances YAP phosphorylation via a negative feedback loop from the Hippo pathway by activating LAST1/2 kinases [42]. Based on the findings of this study and previous functional studies, a proposed molecular interaction between Hippo/YAP and Wnt/β-catenin in promoting colonic tumorigenesis is illustrated in Supplementary Fig. S3. Our findings suggest the above Hippo-independent regulation mechanisms causing YAP deregulation may exist in precancerous cells within human sporadic adenomas.

Significantly, we first reported that elevated p-YAP correlates with the likelihood of large adenomas, villous growth patterns, and advanced adenomas. This correlation is independent of selected variables and other biomarkers, suggesting YAP’s pro-tumorigenic roles in the early stages of sporadic CRC. We also discovered a synergistic interaction between YAP and nuclear β-catenin affecting the odds of advanced adenomas. Our previous research indicated that the Wnt pathway score (the sum of scores for nuclear b-catenin, c-Myc, and Cyclin D1) significantly correlated with larger tumor size, villous growth pattern, and advanced adenomas but not metachronous adenoma [37], which are consistent with this study. Our previous report did not show a significant correlation between nuclear β-catenin and key components of TGF-β, COX-2, or EGFR pathways. Interestingly, this study revealed a synergistic interaction between Wnt/β-catenin and p-YAP influencing the odds of advanced adenomas. As discussed earlier, *APC* mutations coupled with Wnt/β-catenin activation may trigger abnormal elevation of YAP and p-YAP in *APC*-deficient cells. The elevated YAP/p-YAP subsequently promotes β-catenin-driven carcinogenesis by forming YAP-TBX5-β-catenin complex, and phosphorylation of YAP leads to localization at antiapoptotic gene promoters within nuclei [43]. The nuclear accumulation of p-YAP and YAP detected in this study suggests molecular mechanisms for interactions between YAP and Wnt/β-catenin in CRC cells may exist in the benign tumor cells of human sporadic adenomas. Hippo and Wnt are among the top ten major oncogenic signaling pathways likely acting as cancer drivers or therapeutic targets [44]. Our study provides new evidence regarding these two pathways’ synergistic interaction during the early stages of sporadic colorectal carcinogenesis. Furthermore, AUC analysis identified p-YAP as a potential biomarker for distinguishing advanced from nonadvanced adenomas, superior to CtBP and nuclear β-catenin expression.

YAP/TAZ, acting as a bridge for crosstalk with other signaling pathways, modulates activity based on tissue types and cellular contexts. They don’t bind DNA directly but require DNA-binding partners. This has led to their consideration as master transcriptional regulators or an epigenetic “switch” in cancer, enabling phenotypic plasticity and reprogramming the tumor cell ecosystem [16]. The frequent disruption of Hippo-YAP activity may primarily be due to YAP/TAZ’s interaction with other signaling pathways [13,45]. To explore this further, we performed bioinformatics analysis on gene expression data from human adenoma tissues to identify genes and pathways correlated with YAP. We discovered that 543 annotated cancer genes significantly associated with YAP are prevalent in gastrointestinal diseases and play key roles in gene expression, posttranslational modification, protein synthesis, and cell death/survival functions (*P* < .001 for all). The top six enriched signaling pathways among these YAP-correlated genes, especially autophagy, unfolded protein response (UPS), and sirtuin signaling, show a general pro-tumorigenic alteration. The most significantly correlated signaling is autophagy which has been reported as a synergistic interaction with Hippo-YAP in regulating tissue homeostasis and tumorigenesis [46]. The activated autography pathway inhibits YAP via AMP-dependent kinase (AMPK) and Unc-51 like autophagy activating kinase 1 (ULK1), and Hippo/YAP pathway positively or negatively regulate autography via MST1/2, LATS1, and YAP target genes such as Armus, myosin II, p16, and miR-29 [47–49]. Notably, we found that X-box-binding protein 1 (XBP1), one of YAP’s most correlated upstream regulators and a key downstream effector of UPS pathway, may play important roles in human colonic tumorigenesis consistent with other reports [50–52]. Active XBP1 binds to the promoter of β-catenin and activates its expression to indirectly promote YAP expression [53,54]. We found that the key components of sirtuin pathway, Sirtuin 1 (Sirt1) and forkhead box O3 (FOXO3), are significantly associated with the *YAP* gene in promoting colorectal adenomas. Sirt1 promotes Wnt/b-catenin signaling by deacetylating β-catenin and by suppressing Wnt pathway antagonists to activate YAP [55], and YAP, in turn, preserved Sirt1 activity [56]. FOXO3 inhibits the expression and nuclear translocation of β-catenin to suppress β-catenin transcriptional activity [57]. The complex interactions of Hippo-YAP, autophagy, UPS, and Sirtuin pathways are illustrated in Supplementary Fig. S4. The precise molecular interaction of these pathways in promoting colorectal tumorigenesis is still largely unknown. Further research incorporating advanced spatial multiomics and functional *in vitro* studies will be warranted to unveil precise molecular interaction and Hippo-independent deregulation of YAP in its context-dependent pathophysiological roles in colorectal carcinogenesis.

We found a significant correlation between the expressions of p-YAP and CtBP. The latter was notably linked with larger tumor size and villous growth pattern, independently correlating with advanced adenoma. However, CtBP expression levels in adenomas were not significantly higher than those in the normal colonic mucosa of nontumor patients. AUC analysis did not identify it as an effective biomarker for distinguishing advanced from nonadvanced adenomas. A previous study highlighted CtBP’s tumor suppressor functions by direct binding to APC’s C-terminal, reducing free β-catenin availability [28]. In this study, high CtBP expression levels in the normal colonic mucosa of nontumor patients could indicate its tumor suppressor roles in these cells. Yet, in adenoma cells with mutated APC, CtBP can bind to truncated APC’s C-terminal and act as an oncogene, as we observed in this study, via reported oncogenic mechanisms [58]. Further research is required to determine if the strong correlation between p-YAP and CtBP contributes synergistically to sporadic adenoma development.

This study’s strength lies in recruiting participants from a well-established case–control cohort with detailed patient information, rigorous validation of biomarker detection, objective computer-aided imaging quantification, and comprehensive statistical and bioinformatics analysis of *in situ* biomarker data, APC mutations, and gene expression profiling data. However, it does have limitations. The limited sample size may result in nonsignificant associations due to insufficient statistical power. Only eight cases of high-grade dysplasia were available for analysis; these were incorporated into advanced adenomas rather than analyzed separately. This small sample size could also lead to biased positive findings. Despite this, our results are plausible based on previous *in vivo* and *in vitro* studies’ evidence, particularly the consistency of β-catenin results with another adenoma cohort study [37]. Another limitation is that the gene expression data from human adenomas was generated from bulk tumor tissues, which might be compromised due to tissue heterogeneity. While this study showed a significant correlation between elevated p-YAP and deficient-APC/activated Wnt pathway, it couldn’t provide direct evidence of Hippo-independent regulation of YAP via phosphorylation through various pathways or kinases, such as G protein-coupled receptors pathway, protein kinase C zeta, and Rho GTPase [59–61]. Future larger studies integrating more advanced spatial multiomics and functional studies will help confirm YAP’s complex interactions with other cancer pathways to reveal the underlying mechanisms of YAP deregulation and its role in early stage human colorectal carcinogenesis.

## Conclusions

Our research, for the first time, presents new evidence that YAP plays a pro-tumorigenic role in the early stages of human sporadic tumors. Increased p-YAP is linked to *APC* mutations and abnormal WNT/β-catenin pathway, synergistically interacting with nuclear β-catenin for advanced tumor characteristics. Even a slight positivity of elevated p-YAP could be an independent molecular biomarker for sporadic advanced adenomas. We also provide evidence that YAP’s complex crosstalk with autophagy, sirtuin, and unfolded protein response pathways may play roles in colorectal carcinogenesis, and further investigations are warranted.

## Supplementary Material

bgaf007_suppl_Supplementary_Materials

## Data Availability

The data that support the findings of this study are available in the main figures and the supplementary material of this article. Additional supporting data are available from the corresponding authors upon reasonable request.
