# Supplementary material for: Yes-associated protein plays oncogenic roles in human sporadic colorectal adenomas
Source: Carcinogenesis. 2025 Feb 20;46(1):bgaf007. doi: 10.1093/carcin/bgaf007 (PMC11923420; doi:10.1093/carcin/bgaf007)
Supplement: bgaf007_suppl_Supplementary_Materials [file bgaf007_suppl_supplementary_materials.docx]

**Supplementary materials**

**Yes-associated protein (YAP) plays oncogenic roles in sporadic colorectal adenoma**

Lei Fan^1^, Xinyi Guo^1,2^, Mary K Washington^3^, Jiajun Shi^1,2^, Reid M Ness^4^, Qi Liu^5^, Wanqing. Wen^1,2^, Shuya Huang^6^, Xiao Liu^5^, Qiuyin Cai^1,2^, Wei Zheng^1,2^, Robert J Coffey^4,7^, Martha J Shrubsole^1,2*^, Timothy Su^1,2*^

^1^ Division of Epidemiology, Department of Medicine, Vanderbilt Epidemiology Center, Vanderbilt-Ingram Cancer Center, Vanderbilt University Medical Center, Nashville, Tennessee, USA

^2^ GRECC, Department of Veterans Affairs, Tennessee Valley Healthcare System, Nashville, Tennessee, USA

^3^ Department of Pathology, Vanderbilt University Medical Center, Nashville, Tennessee, USA

^4^ Division of Gastroenterology, Department of Medicine, Vanderbilt University Medical Center, Nashville, Tennessee, USA

^5^ Center for Quantitative Sciences and Department of Biostatistics, Vanderbilt University School of Medicine, Nashville, Tennessee, USA

^6^ Department of Breast Surgery, The Second Hospital of Shandong University, Jinan, Shandong, China

^7^ Cell and Development Biology, Vanderbilt University, Nashville, Tennessee, USA

*Correspondence:

Dr. Martha J. Shrubsole, Vanderbilt University Medical Center, 2525 West End Avenue, 8th Floor, Nashville, TN 37203-1738, USA. E-mail: [Martha.Shrubsole@vumc.org](mailto:Martha.Shrubsole@vumc.org)
Dr. Timothy Su, Vanderbilt University Medical Center, 2525 West End Avenue, 8th Floor, Nashville, TN 37203-1738, USA. [Timothy.Su@vumc.org](mailto:Timothy.Su@vumc.org)


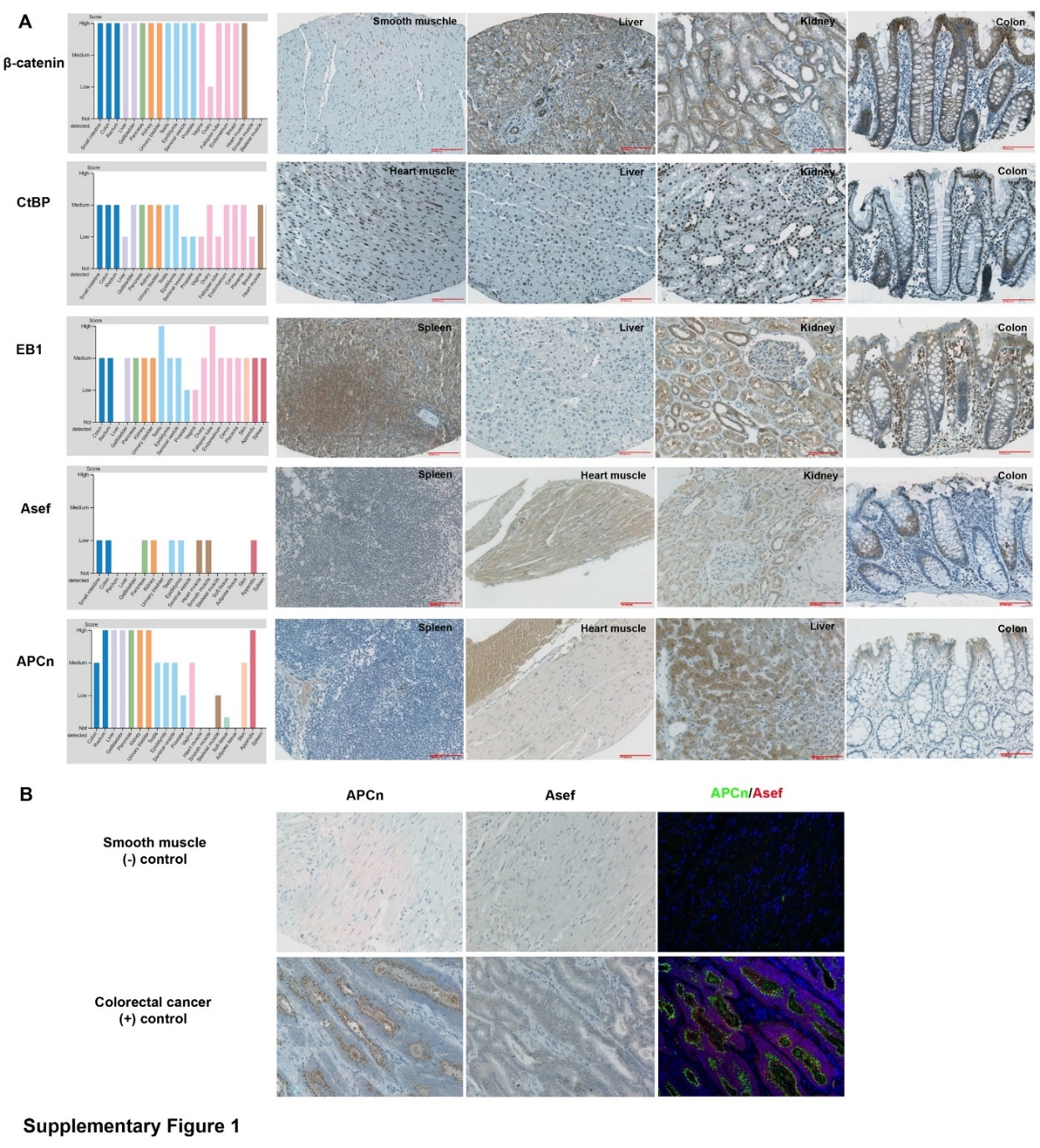


**Supplementary Figure 1. The validation and optimization of staining protocols for all selected biomarkers** **A.** Validation of five primary antibodies using TMA control tissues. The specificity of selected antibodies of β-catenin, CtBP, EB1, Asef, and APCn was confirmed by comparing the staining signals with the antibody-based protein expression data from the Human Protein Atlas (<https://www.proteinatlas.org/about#the_human_protein_atlas>). **B.** A highly sensitive double fluorescence staining for APCn and Asef is validated, showing the same results as the standard IHC methods for each antibody. The smooth muscle and CRC tissue were used as negative and positive control tissues, respectively.


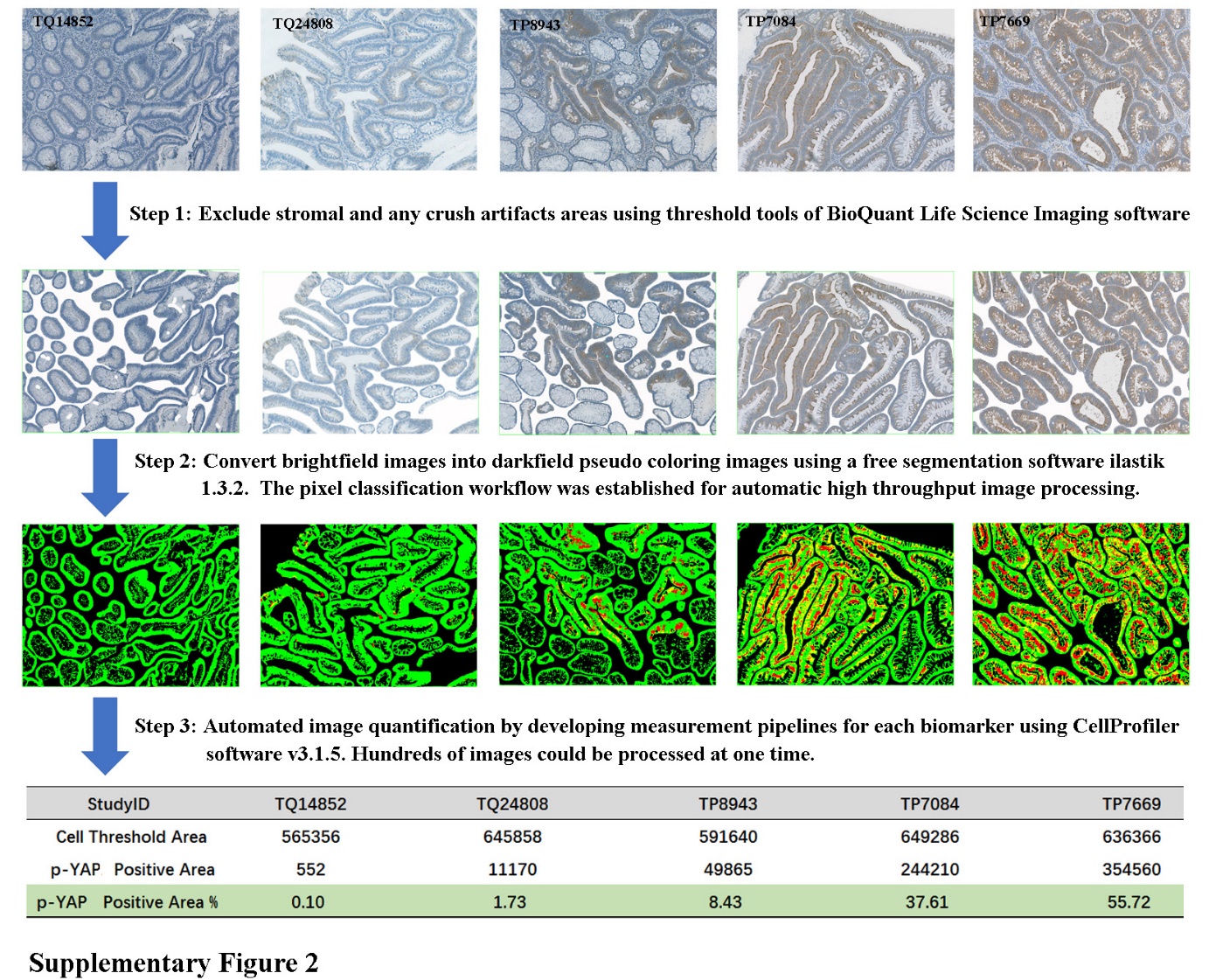


**Supplementary Figure 2. Quantitative analysis workflow of p-YAP as an example** First, images were edited using the threshold tools of BioQuant Life Science imaging software (BioQuant, Nashville, TN) to exclude the stromal area and any unwanted tissue folding area or crush artifacts. Second, the brightfield image was converted into a darkfield pseudo coloring image using free segmentation software ilastik 1.3.2. The pixel classification workflow was established and validated using 20 - 30 reference (TMA controls) and sample images as a training set for machine learning. The tissue components of nuclei, epithelial/tumor cells, and positively stained biomarkers were assigned to blue, green, and red pseudo colors with dark background. The brightfield images were automatically converted into pseudo-coloring images through validated pixel classification pipelines. Last, the automatic imaging quantification of converted pseudo-coloring images was performed by developing measurement pipelines for each biomarker using CellProfiler software v3.1.5. Hundreds of images could be processed at one time and the quantitative data of biomarker expression were exported automatically into Excel files for statistical analysis.

**
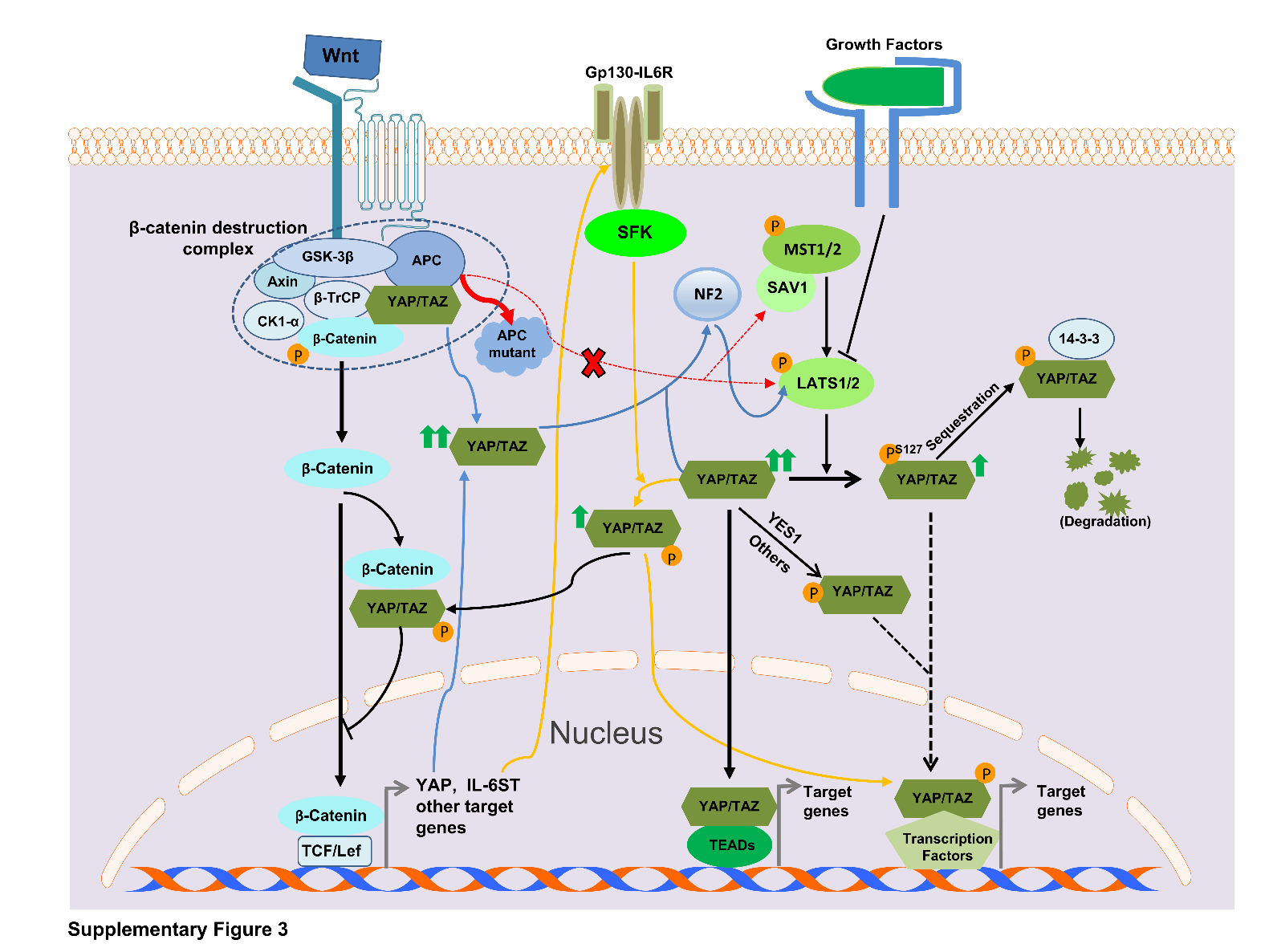
**

**Supplementary Figure 3.** **Crosstalk between activated Wnt/β-catenin** **and Hippo-YAP pathways in promoting human colorectal tumorigenesis.** YAP/TAZ are integral components of the β-catenin destruction complex to recruit β-transducin repeat-containing protein (β-TrCP) for β-catenin degradation (1). In *APC-*mutant cells, YAP is physically dislodged from the complex and causes β-TrCP dissociation from the destruction complex and the release of β-catenin for nuclear translocation to promote *YAP* and other gene expressions (β-catenin-dependent YAP upregulation) (2). APC as a scaffold protein facilitates the phosphorylation of YAP through interacting with the upstream Hippo kinase cascade Sav1 and Lats1, leading to YAP degradation. *APC* mutations cause loss of the function of YAP phosphorylation via APC and therefore activate YAP (β-catenin-independent YAP upregulation) (3,4). Increased YAP interacts with the TEAD/TEF family of transcription factors for YAP-mediated tumor growth (5). The elevated cytoplasmic YAP can activate a “LATS-mediated feedback loop” via NF2 to enhance phosphorylation of YAP (6,7), and activated Wnt/β-catenin signaling promotes IL-6ST/pg130 expression to augment YAP phosphorylation via SFKs activation (7). The cytoplasmic p-YAP, in turn as a negative feedback mechanism, binds to β-catenin to prevent its nuclear translocation (8). YAP can also be phosphorylated by YES1 and other kinases and keeps on entering the nucleus even it is phosphorylated (2,9–13). Nuclear p-YAP binds with other transcription factors to regulate target gene expressions and promote tumorigenesis (7,14). For example, phosphorylation of YAP at tyrosine 357 by YES1 can form a transcription regulator complex of β-catenin-YAP-TBX5 to promote the expression of anti-apoptotic genes (*BCL2L1* and *BIRC5*) to promote tumorigenesis (12).”

**
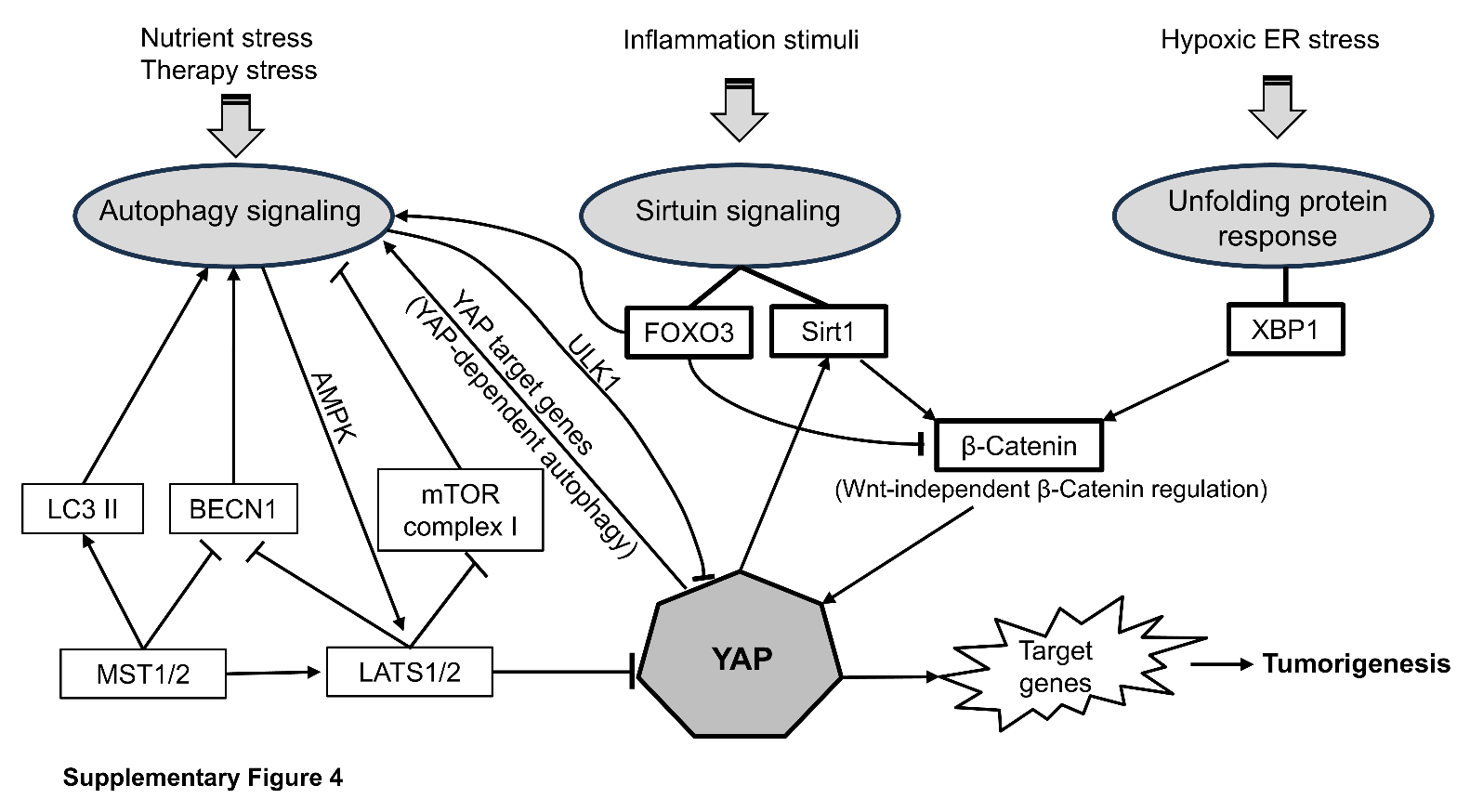
**

**Supplementary Figure S4. Interaction of Hippo-YAP, autophagy, unfolded protein response (UPS), and sirtuin pathways in promoting human colorectal tumorigenesis.** Based on the findings of this study and previous functional studies, we proposed the following interplay mechanisms of Hippo-YAP, autophagy, unfolded protein response (UPS), and sirtuin pathways in promoting human colorectal tumorigenesis. Autophagy signaling was the top enriched pathway among the YAP-correlated genes, showing a general pro-tumorigenic alteration in human colorectal adenoma in the current study. Hippo-YAP pathway positively or negatively regulates autography via MST1/2, LATS1, and YAP target genes (such as Armus, myosin II, p16, and miR-29) under cell context-dependence, and reduced autography weakens its inhibition of YAP via AMP-dependent kinase (AMPK) and Unc-51 like autophagy activating kinase 1 (ULK1) to promote YAP-driven tumorigenesis (15–19). The key component of UPS pathway X-box-binding protein 1 (XBP1), one of YAP's most correlated upstream regulators and a key downstream effector of the UPS pathway, binds to the promoter of β-catenin and activates its expression to promote YAP expression (20,21). The key components of the sirtuin pathway, Sirtuin 1 (Sirt1) and forkhead box O3 (FOXO3), are significantly associated with the *YAP* gene in promoting colorectal adenomas in the current study. Sirt1 promotes Wnt/b-catenin signaling by deacetylating β-catenin and suppressing Wnt pathway antagonists to activate YAP (22), and YAP, in turn, preserves Sirt1 activity (23). FOXO3 inhibits the expression and nuclear translocation of β-catenin to suppress β-catenin transcriptional activity and thus indirectly suppress YAP (24). FOXO3 may also suppress YAP through activating autophagy signaling (25). Therefore, decreased FOXO3, reduced autophagy, and elevated YAP play pro-tumorigenic roles in the human colorectum.

1. Azzolin L, Panciera T, Soligo S *et al*. YAP/TAZ Incorporation in the β-Catenin Destruction Complex Orchestrates the Wnt Response. *Cell*. 2014;158:157-170. doi:10.1016/j.cell.2014.06.013.

2. Konsavage WM, Kyler SL, Rennoll SA *et al*. Wnt/β-Catenin Signaling Regulates Yes-associated Protein (YAP) Gene Expression in Colorectal Carcinoma Cells. *Journal of Biological Chemistry*. 2012;287:11730-11739. doi:10.1074/jbc.M111.327767.

3. Cai J, Maitra A, Anders RA *et al*. β-Catenin destruction complex-independent regulation of Hippo–YAP signaling by APC in intestinal tumorigenesis. *Genes Dev*. 2015;29:1493-1506. doi:10.1101/gad.264515.115.

4. Kim W, Cho YS, Wang X *et al*. Hippo signaling is intrinsically regulated during cell cycle progression by APC/C ^Cdh1^. *Proc Natl Acad Sci USA*. 2019;116:9423-9432. doi:10.1073/pnas.1821370116.

5. Lamar JM, Stern P, Liu H *et al*. The Hippo pathway target, YAP, promotes metastasis through its TEAD-interaction domain. *Proc Natl Acad Sci USA*. 2012;109. doi:10.1073/pnas.1212021109.

6. Moroishi T, Park HW, Qin B *et al*. A YAP/TAZ-induced feedback mechanism regulates Hippo pathway homeostasis. *Genes Dev*. 2015;29:1271-1284. doi:10.1101/gad.262816.115.

7. Taniguchi K, Moroishi T, De Jong PR *et al*. YAP–IL-6ST autoregulatory loop activated on APC loss controls colonic tumorigenesis. *Proc Natl Acad Sci USA*. 2017;114:1643-1648. doi:10.1073/pnas.1620290114.

8. Imajo M, Miyatake K, Iimura A *et al*. A molecular mechanism that links Hippo signalling to the inhibition of Wnt/β-catenin signalling: Hippo signalling regulates β-catenin localization. *The EMBO Journal*. 2012;31:1109-1122. doi:10.1038/emboj.2011.487.

9. Ren F, Zhang L, Jiang J. Hippo signaling regulates Yorkie nuclear localization and activity through 14-3-3 dependent and independent mechanisms. *Developmental Biology*. 2010;337:303-312. doi:10.1016/j.ydbio.2009.10.046.

10. Dupont S, Morsut L, Aragona M *et al*. Role of YAP/TAZ in mechanotransduction. *Nature*. 2011;474:179-183. doi:10.1038/nature10137.

11. Wada K-I, Itoga K, Okano T *et al*. Hippo pathway regulation by cell morphology and stress fibers. *Development*. 2011;138:3907-3914. doi:10.1242/dev.070987.

12. Rosenbluh J, Nijhawan D, Cox AG *et al*. β-Catenin-Driven Cancers Require a YAP1 Transcriptional Complex for Survival and Tumorigenesis. *Cell*. 2012;151:1457-1473. doi:10.1016/j.cell.2012.11.026.

13. Barry ER, Morikawa T, Butler BL *et al*. Restriction of intestinal stem cell expansion and the regenerative response by YAP. *Nature*. 2013;493:106-110. doi:10.1038/nature11693.

14. Werneburg N, Gores GJ, Smoot RL. The Hippo Pathway and YAP Signaling: Emerging Concepts in Regulation, Signaling, and Experimental Targeting Strategies With Implications for Hepatobiliary Malignancies. *gene expr*. 2020;20:67-74. doi:10.3727/105221619X15617324583639.

15. Tang F, Christofori G. The cross-talk between the Hippo signaling pathway and autophagy:implications on physiology and cancer. *Cell Cycle*. 2020;19:2563-2572. doi:10.1080/15384101.2020.1806450.

16. Wang W, Xiao Z-D, Li X *et al*. AMPK modulates Hippo pathway activity to regulate energy homeostasis. *Nat Cell Biol*. 2015;17:490-499. doi:10.1038/ncb3113.

17. Mo J-S, Meng Z, Kim YC *et al*. Cellular energy stress induces AMPK-mediated regulation of YAP and the Hippo pathway. *Nat Cell Biol*. 2015;17:500-510. doi:10.1038/ncb3111.

18. Gan W, Dai X, Dai X *et al*. LATS suppresses mTORC1 activity to directly coordinate Hippo and mTORC1 pathways in growth control. *Nat Cell Biol*. 2020;22:246-256. doi:10.1038/s41556-020-0463-6.

19. Deleyto-Seldas N, Efeyan A. The mTOR–Autophagy Axis and the Control of Metabolism. *Front Cell Dev Biol*. 2021;9:655731. doi:10.3389/fcell.2021.655731.

20. Xia Z, Wu S, Wei X *et al*. Hypoxic ER stress suppresses β-catenin expression and promotes cooperation between the transcription factors XBP1 and HIF1α for cell survival. *Journal of Biological Chemistry*. 2019;294:13811-13821. doi:10.1074/jbc.RA119.008353.

21. Shi W, Chen Z, Li L *et al*. Unravel the molecular mechanism of XBP1 in regulating the biology of cancer cells. *J Cancer*. 2019;10:2035-2046. doi:10.7150/jca.29421.

22. O’Callaghan C, Vassilopoulos A. Sirtuins at the crossroads of stemness, aging, and cancer. *Aging Cell*. 2017;16:1208-1218. doi:10.1111/acel.12685.

23. Yan H, Qiu C, Sun W *et al*. Yap regulates gastric cancer survival and migration via SIRT1/Mfn2/mitophagy. *Oncol Rep*. February 2018. doi:10.3892/or.2018.6252.

24. Liu H, Yin J, Wang H *et al*. FOXO3a modulates WNT/β-catenin signaling and suppresses epithelial-to-mesenchymal transition in prostate cancer cells. *Cellular Signalling*. 2015;27:510-518. doi:10.1016/j.cellsig.2015.01.001.

25. Zhao J, Brault JJ, Schild A *et al*. FoxO3 Coordinately Activates Protein Degradation by the Autophagic/Lysosomal and Proteasomal Pathways in Atrophying Muscle Cells. *Cell Metabolism*. 2007;6:472-483. doi:10.1016/j.cmet.2007.11.004.

| **Supplementary Table 1. Association of biomarker expression with clinicopathological parameters of adenomas** | | | | | | | | | | | | | | | | | | | | | | | | | | | | |
| --- | --- | --- | --- | --- | --- | --- | --- | --- | --- | --- | --- | --- | --- | --- | --- | --- | --- | --- | --- | --- | --- | --- | --- | --- | --- | --- | --- | --- |
| **Genetic variables** | **Tumor size** | | | | | | | | |  |  | **Histotype** | | | |  |  | **Advanced adenoma** | | | |  |  | **Metachronous adenoma** | | | |  |
|  | **<4mm** | **4 to < 10mm, OR (95%CI)** | | |  |  | **>=10mm, OR (95%CI)** | | |  |  | **TA** | **TV/V** | **OR (95%CI)** | |  |  | **No** | **Yes** | **OR (95%CI)** | |  |  | **No** | **Yes** | **OR (95%CI)** | |  |
|  | **n** | **n** | **Model 1** | **Model 2** | **Model 3** |  | **n** | **Model 1** | **Model 2** | **Model 3** |  | **n** | **n** | **Model 1** | **Model 2** | **Model 3** |  | **n** | **n** | **Model 1** | **Model 2** | **Model 3** |  | **n** | **n** | **Model 1** | **Model 2** | **Model 3** |
| β-catenin |  |  |  |  |  |  |  |  |  |  |  |  |  |  |  |  |  |  |  |  |  |  |  |  |  |  |  |  |
| 0.41-20.71 | 11 | 10 | 1.00 (ref) | 1.00 (ref) | 1.00 (ref) |  | 12 | 1.00(ref) | 1.00(ref) | 1.00 (ref) |  | 24 | 9 | 1.00(ref) | 1.00(ref) | 1.00(ref) |  | 17 | 16 | 1.00(ref) | 1.00(ref) | 1.00(ref) |  | 12 | 21 | 1.00(ref) | 1.00(ref) | 1.00(ref) |
| 20.71-43.44 | 22 | 8 | 0.40 (0.12-1.30) | 0.36 (0.11-1.22) | 0.25 (0.06-1.11) |  | 14 | 0.58 (0.20-1.68) | 0.68 (0.23-1.04) | 0.31 (0.07-1.35) |  | 33 | 11 | 0.89 (0.32-2.48) | 1.08 (0.37-3.14) | 0.48 (0.11-2.11) |  | 28 | 16 | 0.61 (0.24-1.52) | 0.71 (0.27-1.83) | 0.40 (0.11-1.40) |  | 29 | 15 | 0.30 (0.12-0.76) | 0.35 (0.13-0.94) | 0.29 (0.09-0.97) |
| 43.44-84.72 | 31 | 12 | 0.43 (0.14-1.26) | 0.41 (0.13-1.24) | 0.15 (0.03-0.77) |  | 23 | 0.68 (0.26-1.81) | 0.76 (0.28-2.10) | 0.14 (0.03-0.73) |  | 48 | 18 | 1.00 (0.39-2.56) | 1.01 (0.38-2.67) | 0.01 (<0.001-0.18) |  | 38 | 28 | 0.78 (0.34-1.81) | 0.87 (0.37-2.07) | 0.09 (0.02-0.47) |  | 36 | 30 | 0.48 (0.20-1.12) | 0.57 (0.24-1.38) | 0.73 (0.20-2.63) |
| *P* trend |  |  | 0.1668 | 0.1641 | 0.0234 |  |  | 0.5564 | 0.7045 | 0.022 |  |  |  | 0.9565 | 0.9949 | 0.0013 |  |  |  | 0.6956 | 0.8605 | 0.0039 |  |  |  | 0.1926 | 0.3765 | 0.8267 |
|  |  |  |  |  |  |  |  |  |  |  |  |  |  |  |  |  |  |  |  |  |  |  |  |  |  |  |  |  |
| Nuclear β-catenin |  |  |  |  |  |  |  |  |  |  |  |  |  |  |  |  |  |  |  |  |  |  |  |  |  |  |  |  |
| 0 | 35 | 17 | 1.00 (ref) | 1.00 (ref) | 1.00 (ref) |  | 23 | 1.00 (ref) | 1.00 (ref) | 1.00 (ref) |  | 60 | 15 | 1.00(ref) | 1.00(ref) | 1.00(ref) |  | 46 | 29 | 1.00(ref) | 1.00(ref) | 1.00(ref) |  | 39 | 36 | 1.00(ref) | 1.00(ref) | 1.00(ref) |
| 1 | 13 | 4 | 0.63 (0.18-2.24) | 0.61 (0.173-2.19) | 0.49 (0.09-2.68) |  | 8 | 0.94 (0.34-2.61) | 0.83 (0.29-2.41) | 0.54 (0.13-2.26) |  | 17 | 8 | 1.88 (0.68-5.18) | 2.51 (0.85-7.43) | 6.88 (1.36-34.89) |  | 14 | 11 | 1.25 (0.50-3.12) | 1.17 (0.46-3.01) | 1.02 (0.29-3.58) |  | 14 | 11 | 0.85 (0.34-2.12) | 0.73 (0.28-1.89) | 0.67 (0.22-2.10) |
| 2 | 12 | 7 | 1.20 (0.40-3.60) | 1.31 (0.42-4.05) | 0.92 (0.20-4.25) |  | 7 | 0.89 (0.30-2.59) | 0.94 (0.31-2.82) | 0.42 (0.08-2.22) |  | 19 | 7 | 1.47 (0.52-4.15) | 1.86 (0.62-5.52) | 1.62 (0.31-8.56) |  | 17 | 9 | 0.84 (0.33-2.13) | 0.88 (0.34-2.28) | 0.41 (0.09-1.89) |  | 16 | 10 | 0.68 (0.27-1.68) | 0.65 (0.25-1.68) | 0.52 (0.16-1.72) |
| 3 | 5 | 2 | 0.82 (0.15-4.69) | 0.85 (0.15-4.90) | 1.11 (0.12-10.51) |  | 11 | 3.35 (1.03-10.90) | 2.88 (0.85-9.77) | 3.61 (0.66-19.85) |  | 10 | 8 | 3.20 (1.08-9.50) | 3.91 (1.21-12.60) | 2.69 (0.47-15.59) |  | 7 | 11 | 2.49 (0.87-7.16) | 2.12 (0.71-6.32) | 1.89 (0.42-8.62) |  | 9 | 9 | 1.08 (0.39-3.03) | 0.81 (0.27-2.44) | 0.84 (0.20-3.45) |
| *P* trend |  |  | 1.00 | 0.9196 | 0.9828 |  |  | 0.1246 | 0.1867 | 0.4935 |  |  |  | 0.0507 | 0.025 | 0.2743 |  |  |  | 0.2527 | 0.3699 | 0.8969 |  |  |  | 0.7551 | 0.4618 | 0.4458 |
|  |  |  |  |  |  |  |  |  |  |  |  |  |  |  |  |  |  |  |  |  |  |  |  |  |  |  |  |  |
| Nuclear β-catenin |  |  |  |  |  |  |  |  |  |  |  |  |  |  |  |  |  |  |  |  |  |  |  |  |  |  |  |  |
| 0 | 35 | 17 | 1.00(ref) | 1.00(ref) | 1.00(ref) |  | 23 | 1.00(ref) | 1.00(ref) | 1.00(ref) |  | 60 | 15 | 1.00(ref) | 1.00(ref) | 1.00(ref) |  | 46 | 29 | 1.00(ref) | 1.00(ref) | 1.00(ref) |  | 39 | 36 | 1.00(ref) | 1.00(ref) | 1.00(ref) |
| 1-3 | 30 | 13 | 0.89 (0.37-2.13) | 0.91 (0.37-2.23) | 0.78 (0.24-2.50) |  | 26 | 1.32 (0.63-2.77) | 1.23 (0.57-2.67) | 0.86 (0.30-2.47) |  | 46 | 23 | 2.00 (0.94-4.26) | 2.54 (1.12-5.74) | 3.09 (0.93-10.22) |  | 38 | 31 | 1.29 (0.67-2.51) | 1.22 (0.61-2.44) | 0.92 (0.35-2.40) |  | 39 | 30 | 0.83 (0.43-1.61) | 0.72 (0.36-1.44) | 0.65 (0.27-1.54) |
| *P* trend |  |  | 0.7974 | 0.8423 | 0.6761 |  |  | 0.4656 | 0.6043 | 0.7761 |  |  |  | 0.0721 | 0.0251 | 0.0648 |  |  |  | 0.4468 | 0.5676 | 0.8574 |  |  |  | 0.5866 | 0.3521 | 0.3233 |
|  |  |  |  |  |  |  |  |  |  |  |  |  |  |  |  |  |  |  |  |  |  |  |  |  |  |  |  |  |
| EB1 |  |  |  |  |  |  |  |  |  |  |  |  |  |  |  |  |  |  |  |  |  |  |  |  |  |  |  |  |
| 0.02-2.91 | 21 | 12 | 1.00(ref) | 1.00(ref) | 1.00(ref) |  | 10 | 1.00(ref) | 1.00(ref) | 1.00(ref) |  | 36 | 7 | 1.00(ref) | 1.00(ref) | 1.00(ref) |  | 30 | 13 | 1.00(ref) | 1.00(ref) | 1.00(ref) |  | 24 | 19 | 1.00(ref) | 1.00(ref) | 1.00(ref) |
| 2.91-8.64 | 22 | 9 | 0.72 (0.25-2.05) | 0.71 (0.24-2.04) | 0.48 (0.12-1.90) |  | 17 | 1.62 (0.61-4.34) | 2.01 (0.71-5.68) | 1.06 (0.27-4.09) |  | 32 | 16 | 2.57 (0.94-7.04) | 2.89 (0.98-8.47) | 1.90 (0.42-8.61) |  | 26 | 22 | 1.95 (0.82-4.63) | 2.29 (0.93-5.64) | 1.35 (0.40-4.57) |  | 22 | 26 | 1.49 (0.65-3.42) | 1.48 (0.63-3.48) | 1.67 (0.59-4.76) |
| 8.64-82.9 | 21 | 9 | 0.75 (0.26-2.15) | 0.75 (0.26-2.19) | 0.63 (0.16-2.51) |  | 23 | 2.30 (0.88-5.99) | 3.03 (1.09-8.42) | 1.42 (0.37-5.52) |  | 37 | 16 | 2.22 (0.82-6.04) | 2.63 (0.90-7.66) | 1.23 (0.28-5.48) |  | 27 | 26 | 2.22 (0.96-5.17) | 2.74 (1.13-6.66) | 1.23 (0.36-4.18) |  | 31 | 22 | 0.90 (0.40-2.02) | 0.96 (0.41-2.23) | 0.84 (0.30-2.41) |
| *P* trend |  |  | 0.5784 | 0.5809 | 0.4854 |  |  | 0.0906 | 0.0353 | 0.6394 |  |  |  | 0.1495 | 0.1016 | 0.7951 |  |  |  | 0.071 | 0.0305 | 0.7454 |  |  |  | 0.7344 | 0.8611 | 0.7305 |
|  |  |  |  |  |  |  |  |  |  |  |  |  |  |  |  |  |  |  |  |  |  |  |  |  |  |  |  |  |
| CtBP |  |  |  |  |  |  |  |  |  |  |  |  |  |  |  |  |  |  |  |  |  |  |  |  |  |  |  |  |
| 0-4.61 | 30 | 11 | 1.00(ref) | 1.00(ref) | 1.00(ref) |  | 14 | 1.00(ref) | 1.00(ref) | 1.00(ref) |  | 49 | 6 | 1.00(ref) | 1.00(ref) | 1.00(ref) |  | 40 | 15 | 1.00(ref) | 1.00(ref) | 1.00(ref) |  | 26 | 29 | 1.00(ref) | 1.00(ref) | 1.00(ref) |
| 4.61-15.32 | 17 | 11 | 1.77 (0.63-4.92) | 1.81 (0.63-5.19) | 1.70 (0.46-6.36) |  | 8 | 1.01 (0.35-2.89) | 0.83 (0.27-2.49) | 0.86 (0.20-3.62) |  | 27 | 9 | 2.72 (0.88-8.46) | 2.45 (0.77-7.82) | 1.38 (0.28-6.88) |  | 22 | 14 | 1.70 (0.69-4.15) | 1.47 (0.59-3.69) | 1.99 (0.56-7.08) |  | 18 | 18 | 0.90 (0.39-2.08) | 0.73 (0.30-1.75) | 0.75 (0.26-2.21) |
| 15.32-93.59 | 18 | 8 | 1.21 (0.41-3.58) | 1.31 (0.44-3.93) | 1.08 (0.18-6.29) |  | 27 | 3.21 (1.35-7.68) | 3.20 (1.31-7.83) | 3.60 (0.77-16.76) |  | 29 | 24 | 6.76 (2.47-18.47) | 6.11 (2.19-17.04) | 11.62 (1.98-68.19) |  | 22 | 31 | 3.76 (1.68-8.42) | 3.67 (1.61-8.37) | 5.27 (1.25-22.23) |  | 34 | 19 | 0.50 (0.23-1.08) | 0.46 (0.21-1.04) | 0.66 (0.21-2.14) |
| *P* trend |  |  | 0.6522 | 0.5641 | 0.6966 |  |  | 0.0068 | 0.0086 | 0.1388 |  |  |  | 0.0001 | 0.0004 | 0.0103 |  |  |  | 0.0013 | 0.002 | 0.0232 |  |  |  | 0.0809 | 0.0615 | 0.4695 |
|  |  |  |  |  |  |  |  |  |  |  |  |  |  |  |  |  |  |  |  |  |  |  |  |  |  |  |  |  |
| Asef |  |  |  |  |  |  |  |  |  |  |  |  |  |  |  |  |  |  |  |  |  |  |  |  |  |  |  |  |
| 0-0.88 | 27 | 2 | 1.00 (ref) | 1.00 (ref) | 1.00 (ref) |  | 3 | 1.00 (ref) | 1.00 (ref) | 1.00 (ref) |  | 29 | 3 | 1.00(ref) | 1.00(ref) | 1.00(ref) |  | 27 | 5 | 1.00(ref) | 1.00(ref) | 1.00(ref) |  | 13 | 19 | 1.00(ref) | 1.00(ref) | 1.00(ref) |
| 0.88-6.92 | 21 | 17 | 10.93 (2.27-52.64) | 10.67 (2.18-52.19) | 6.79 (1.09-42.27) |  | 28 | 11.99 (3.20-44.90) | 11.48 (3.01-43.77) | 22.04 (2.13-228.11) |  | 44 | 22 | 4.83 (1.33-17.63) | 5.70 (1.53-21.27) | 3.16 (0.46-21.62) |  | 31 | 35 | 6.10 (2.09-17.77) | 6.06 (2.04-18.01) | 9.94 (1.56-63.28) |  | 34 | 32 | 0.64 (0.27-1.51) | 0.55 (0.22-1.35) | 0.66 (0.20-2.23) |
| 6.92-98.18 | 17 | 11 | 8.74 (1.72-44.32) | 8.34 (1.63-42.75) | 6.28 (0.94-41.88) |  | 19 | 10.06 (2.58-39.19) | 9.48 (2.40-37.50) | 15.29 (1.37-170.64) |  | 33 | 14 | 4.10 (1.07-15.71) | 4.17 (1.06-16.39) | 2.36 (0.31-18.18) |  | 26 | 21 | 4.36 (1.43-13.29) | 4.20 (1.36-13.03) | 5.96 (0.88-40.39) |  | 31 | 16 | 0.35 (0.14-0.89) | 0.33 (0.13-0.87) | 0.27 (0.07-0.98) |
| *P* trend |  |  | 0.0059 | 0.0086 | 0.0686 |  |  | 0.0008 | 0.0015 | 0.053 |  |  |  | 0.077 | 0.0887 | 0.6048 |  |  |  | 0.0275 | 0.0381 | 0.2523 |  |  |  | 0.0253 | 0.0251 | 0.0307 |
|  |  |  |  |  |  |  |  |  |  |  |  |  |  |  |  |  |  |  |  |  |  |  |  |  |  |  |  |  |
| p-YAP |  |  |  |  |  |  |  |  |  |  |  |  |  |  |  |  |  |  |  |  |  |  |  |  |  |  |  |  |
| 0-0.08 | 19 | 6 | 1.00(ref) | 1.00(ref) | 1.00(ref) |  | 7 | 1.00(ref) | 1.00(ref) | 1.00(ref) |  | 74 | 3 | 1.00(ref) | 1.00(ref) | 1.00(ref) |  | 63 | 14 | 1.00(ref) | 1.00(ref) | 1.00(ref) |  | 19 | 13 | 1.00(ref) | 1.00(ref) | 1.00(ref) |
| 0.08-0.69 | 35 | 5 | 0.45 (0.12-1.68) | 0.39 (0.10-1.52) | 0.97 (0.15-6.39) |  | 5 | 0.39 (0.11-1.39) | 0.27 (0.07-1.12) | 0.52 (0.10-2.71) |  |  |  |  |  |  |  |  |  |  |  |  |  | 25 | 20 | 1.17 (0.47-2.93) | 1.06 (0.41-2.77) | 0.89 (0.26-3.07) |
| 0.69-55.72 | 11 | 19 | 5.47 (1.68-17.81) | 5.63 (1.69-18.82) | 23.29 (3.23-168.09) |  | 37 | 9.13 (3.05-27.35) | 9.53 (3.08-29.50) | 19.38 (3.21-117.16) |  | 31 | 36 | 28.65 (8.21-100.00) | 29.48 (8.19-106.11) | 99.60 (12.08-821.54) |  | 21 | 46 | 9.86 (4.54-21.41) | 11.09 (4.90-25.07) | 12.31 (3.78-40.10) |  | 33 | 34 | 1.51 (0.64-3.53) | 1.54 (0.64-3.69) | 1.52 (0.46-5.05) |
| *P* trend |  |  | 0.0025 | 0.0025 | 0.0007 |  |  | <.0001 | <.0001 | 0.0003 |  |  |  | <.0001 | <.0001 | <.0001 |  |  |  | <.0001 | <.0001 | <.0001 |  |  |  | 0.3213 | 0.2873 | 0.4062 |
|  |  |  |  |  |  |  |  |  |  |  |  |  |  |  |  |  |  |  |  |  |  |  |  |  |  |  |  |  |
| APCn |  |  |  |  |  |  |  |  |  |  |  |  |  |  |  |  |  |  |  |  |  |  |  |  |  |  |  |  |
| 0-2.31 | 19 | 8 | 1.00(ref) | 1.00(ref) | 1.00(ref) |  | 9 | 1.00(ref) | 1.00(ref) | 1.00(ref) |  | 25 | 11 | 1.00(ref) | 1.00(ref) | 1.00(ref) |  | 22 | 14 | 1.00(ref) | 1.00(ref) | 1.00(ref) |  | 17 | 19 | 1.00(ref) | 1.00(ref) | 1.00(ref) |
| 2.31-12.85 | 23 | 14 | 1.45 (0.50-4.17) | 1.30 (0.44-3.85) | 0.61 (0.12-3.08) |  | 25 | 2.29 (0.87-6.08) | 2.35 (0.86-6.40) | 1.77 (0.37-8.36) |  | 46 | 16 | 0.79 (0.32-1.96) | 0.89 (0.35-2.27) | 0.20 (0.04-1.10) |  | 33 | 29 | 1.38 (0.60-3.18) | 1.42 (0.60-3.34) | 1.19 (0.31-4.53) |  | 31 | 31 | 0.90 (0.39-2.04) | 0.96 (0.41-2.25) | 1.23 (0.40-3.76) |
| 12.85-98.55 | 23 | 8 | 0.83 (0.26-2.62) | 0.74 (0.23-2.43) | 0.17 (0.03-1.06) |  | 16 | 1.47 (0.53-4.06) | 1.44 (0.50-4.13) | 0.42 (0.08-2.28) |  | 35 | 12 | 0.78 (0.30-2.05) | 0.87 (0.31-2.40) | 0.34 (0.06-1.96) |  | 29 | 18 | 0.98 (0.40-2.38) | 0.97 (0.38-2.44) | 0.42 (0.10-1.80) |  | 30 | 17 | 0.51 (0.21-1.23) | 0.59 (0.23-1.46) | 0.57 (0.16-2.04) |
| *P* trend |  |  | 0.7116 | 0.5894 | 0.0515 |  |  | 0.5789 | 0.6248 | 0.2586 |  |  |  | 0.6263 | 0.7873 | 0.2192 |  |  |  | 0.8885 | 0.878 | 0.231 |  |  |  | 0.1189 | 0.2312 | 0.3987 |
| Model 1: unadjusted model. Model 2: models were partially adjusted for age, sex, and race. Model 3: model 2 additionally mutually adjusted for biomarkers with P<0.20 in crude models.  * Biomarkers were presented with two tertiles combined for low versus high values due to the small sample size. | | | | | | | | | | | | | | | | | | | | | | | | | | | | |

| **Supplementary Table 2. Association between APC mutation characteristics and metachronous adenoma risk** | | | | | |
| --- | --- | --- | --- | --- | --- |
| **Mutation status** | **Baseline adenoma** | **Metachronous adenoma*** | **Metachronous-matched baseline** | **P1** | **P2** |
| No | 51(25.5) | 25 (50.0) | 9 (18.0) | 0.8711 | 0.0007 |
| Yes | 149 (74.5) | 25 (50.0) | 41 (82.0) |  |  |
| **Mutation site** |  |  |  |  |  |
| 0. No mutation | 51 (25.5) | 25 (50.0) | 9 (18.0) | ref | ref |
| 1. Exons 1 to part of 14 (0-1018aa) | 27 (13.5) | 4 (8.0) | 5 (10.0) | 0.7003 | 0.0978 |
| 2. Exon 14 (1019-1600aa) | 59 (29.5) | 10 (20.0) | 17 (34.0) | 0.8701 | 0.0042 |
| 3. Regions 1 + 2 | 62 (31.0) | 10 (20.0) | 19 (38.0) | 0.4308 | 0.0019 |
| **Mutation multiplicity** |  |  |  |  |  |
| 0. No mutation | 51 (25.5) | 25 (50.0) | 9 (18.0) | ref | ref |
| 1. Single mutation | 74 (37.0) | 12 (24.0) | 19 (38.0) | 0.7351 | 0.0046 |
| 2. Double mutations | 65 (32.5) | 9 (18.0) | 20 (40.0) | 0.4956 | 0.0007 |
| 3. >2 mutations | 9 (4.5) | 3 (6.0) | 2 (4.0) | 0.7177 | 0.5302 |
| **Mutation types** |  |  |  |  |  |
| 0. No mutation | 51 (25.5) | 25 (50.0) | 9 (18.0) | ref | ref |
| 1. Stopgain only | 63 (31.5) | 11 (22.0) | 12 (24.0) | 0.6263 | 0.0484 |
| 2. Frameshift**** | 44 (22.0) | 8 (16.0) | 14 (28.0) | 0.9 | 0.0058 |
| 3. 1+2 | 41 (20.5) | 5 (10.0) | 15 (30.0) | 0.1674 | 0.0005 |
| Total | 199** | 49*** | 50 |  |  |
| *The metachronous adenomas are case-matched with baseline adenomas. | | | |  |  |
| **Exclude one case (TQ15338) of missense mutation at exon 3 (R109W). | | | |  |  |
| ***Exclude one case (TP1145) of missense mutation at exon14. ****Framshift mutation includes deletion, insertion, and substitution. | | | | | |
| P1: Baseline synchronous (100 cases) vs. baseline metachronous (100 cases), not case matched. | | | | | |
| P2: Metachronous vs. matched baseline adenomas (50 cases-matched samples).           \| **Supplementary Table 3. Association of biomarker expression with APC mutation status in adenomas*** \| \| \| \| \| \| \| \| \| \| \| \| \| \| \| \| \| \| \| \| \| \| \| \| --- \| --- \| --- \| --- \| --- \| --- \| --- \| --- \| --- \| --- \| --- \| --- \| --- \| --- \| --- \| --- \| --- \| --- \| --- \| --- \| --- \| --- \| --- \| \| **APC mutation status** \| **Case** \| **Nuclear β-catenin**  **n (%)** \| \|  \|  \| **β-Catenin** \| \|  \| **p-YAP** \| \|  \| **EB1** \| \|  \| **CtBP** \| \|  \| **Asef** \| \|  \| **APCn** \| \| \|  \| n \| Negative \| Positive \| *P* \|  \| mean (sd) \| *P* \|  \| mean (sd) \| *P* \|  \| mean (sd) \| *P* \|  \| mean (sd) \| *P* \|  \| mean (sd) \| *P* \|  \| mean (sd) \| *P* \| \| **Mutation status** \|  \|  \|  \|  \|  \|  \|  \|  \|  \|  \|  \|  \|  \|  \|  \|  \|  \|  \|  \|  \|  \|  \| \| No \| 19 \| 14 (24.1) \| 5 (10.0) \| 0.054 \|  \| 43.1 (24.0) \| 0.493** \|  \| 2.5 (5.2) \| 0.05** \|  \| 7.0 (7.3) \| 0.427** \|  \| 13.2 (20.6) \| 0.123** \|  \| 13 (20.3) \| 0.794** \|  \| 17.3 (24.9) \| 0.065** \| \| Yes \| 89 \| 44 (75.9) \| 45 (90.0) \|  \| 40.7 (21.4) \|  \| 6.7 (10.7) \|  \| 12.6 (17.1) \|  \| 17.5 (17.7) \|  \| 8.7 (12.9) \|  \| 10.4 (12.8) \| \| **APC mutation sites** \|  \|  \|  \|  \|  \|  \|  \|  \|  \|  \|  \|  \|  \|  \|  \|  \|  \|  \|  \|  \|  \|  \| \| No mutation \| 19 \| 14 (24.1) \| 5 (10.0) \|  \|  \| 43.1 (24.0) \|  \|  \| 2.5 (5.2) \|  \|  \| 7.0 (7.3) \|  \|  \| 13.2 (20.6) \|  \|  \| 13 (20.3) \|  \|  \| 17.3 (24.9) \|  \| \| Region 1 (0-1018aa) \| 14 \| 5 (8.6) \| 9 (18.0) \| 0.167 \|  \| 50.6 (25.1) \| 0.220 \|  \| 7.2 (10.4) \| 0.424 \|  \| 12.0 (12.7) \| 0.535 \|  \| 26.1 (26.9) \| 0.135 \|  \| 12.2 (13.5) \| 0.324 \|  \| 10.1 (10.5) \| 0.384 \| \| Region 2 (1019-1600aa) \| 36 \| 18 (31.0) \| 18 (36.0) \|  \| 41.2 (19.5) \|  \| 6.1 (11.3) \|  \| 13.7 (20.5) \|  \| 16.6 (15.6) \|  \| 9.6 (17.2) \|  \| 10.4 (16.2) \| \| Regions 1+2 \| 39 \| 21 (36.2) \| 18 (36.0) \|  \| 36.7 (21.0) \|  \| 7.0 (10.4) \|  \| 11.7 (15.1) \|  \| 15.2 (14.7) \|  \| 6.7 (6.9) \|  \| 10.5 (10.1) \| \| **APC mutation multiplicity** \| \|  \|  \|  \|  \|  \|  \|  \|  \|  \|  \|  \|  \|  \|  \|  \|  \|  \|  \|  \|  \|  \| \| No mutation \| 19 \| 14 (24.1) \| 5 (10.0) \|  \|  \| 43.1 (24.0) \|  \|  \| 2.5 (5.2) \|  \|  \| 7.0 (7.3) \|  \|  \| 13.2 (20.6) \|  \|  \| 13 (20.3) \|  \|  \| 17.3 (24.9) \|  \| \| Single mutation \| 41 \| 17 (29.3) \| 24 (48.0) \| 0.093 \|  \| 42.6 (21.8) \| 0.612 \|  \| 5.0 (8.4) \| 0.139 \|  \| 11.9 (16.0) \| 0.245 \|  \| 16.9 (18.9) \| 0.632 \|  \| 10.4 (16.6) \| 0.561 \|  \| 9.5 (13.4) \| 0.308 \| \| Double mutations \| 41 \| 22 (37.9) \| 19 (38.0) \|  \| 40.4 (20.3) \|  \| 8.6 (12.7) \|  \| 11.7 (16.3) \|  \| 18.8 (17.2) \|  \| 6.9 (8.9) \|  \| 10.7 (12.8) \| \| More than 2 mutations \| 7 \| 5 (8.6) \| 2 (4.0) \|  \| 31.2 (25.5) \|  \| 4.7 (8.5) \|  \| 21.3 (26.6) \|  \| 12.7 (14.4) \|  \| 9.6 (6.4) \|  \| 14.1 (10.2) \| \| **APC mutation types***** \| \|  \|  \|  \|  \|  \|  \|  \|  \|  \|  \|  \|  \|  \|  \|  \|  \|  \|  \|  \|  \|  \| \| No mutation \| 19 \| 14 (24.1) \| 5(10.0) \|  \|  \| 43.1 (24.0) \|  \|  \| 2.5 (5.2) \|  \|  \| 7.0 (7.3) \|  \|  \| 13.2 (20.6) \|  \|  \| 13 (20.3) \|  \|  \| 17.3 (24.9) \|  \| \| Stopgain only \| 37 \| 16 (27.6) \| 21 (42.0) \| 0.197 \|  \| 39.1 (21.6) \| 0.643 \|  \| 6.7 (10.9) \| 0.238 \|  \| 9.8 (10.6) \| 0.231 \|  \| 14.5 (15.9) \| 0.499 \|  \| 9 (11.4) \| 0.648 \|  \| 11.8 (13.0) \| 0.422 \| \| Frameshift only \| 25 \| 13 (22.4) \| 12 (24.0) \|  \| 42.2 (20.7) \|  \| 4.6 (5.1) \|  \| 13.4 (19.2) \|  \| 21.9 (18.8) \|  \| 11.2 (17.2) \|  \| 9.0 (11.1) \| \| Both \| 27 \| 15 (25.9) \| 12 (24.0) \|  \| 40.4 (22.0) \|  \| 8.9 (14.1) \|  \| 14.8 (21.7) \|  \| 17 (18.9) \|  \| 6.3 (9.9) \|  \| 10.2 (14.5) \| \| *Chi-square test for categorical data and ANOVA for continuous data. **Wilcoxon rank sum test for continuous data of two groups. ***Frameshift includes deletion, insertion, and substitution mutations. Nonsynonymous SNV exists alone in 1 case which was excluded from the analysis. In the other 7 cases, nonsynonymous SNV co-exists with stopgain (3 cases) was assigned to "Stopgain only" group; nonsynonymous SNV co-exists with frameshift deletion (1 case) was assigned to Frameshift only" group; nonsynonymous SNV co-exists with both frameshift deletion and stopgain (2 cases) or splicing (1 case) was assigned to "Both" groups. One case (TP1626) failed in β-catenin staining. \| \| \| \| \| \| \| \| \| \| \| \| \| \| \| \| \| \| \| \| \| \| \| \|  \| | | | | | |
